# Mitochondrial genome diversity in dagger and needle nematodes (Nematoda: Longidoridae)

**DOI:** 10.1038/srep41813

**Published:** 2017-02-02

**Authors:** J. E. Palomares-Rius, C. Cantalapiedra-Navarrete, A. Archidona-Yuste, V. C. Blok, P. Castillo

**Affiliations:** 1 Instituto de Agricultura Sostenible (IAS), Agencia Estatal Consejo Superior de Investigaciones Científicas (CSIC), Avda. Menéndez Pidal s/n, 14004 Córdoba, Spain; 2 Cell and Molecular Sciences, The James Hutton Institute, Invergowrie, Dundee, DD2 5DA, United Kingdom

## Abstract

Dagger and needle nematodes included in the family Longidoridae (*viz. Longidorus, Paralongidorus*, and *Xiphinema*) are highly polyphagous plant-parasitic nematodes in wild and cultivated plants and some of them are plant-virus vectors (nepovirus). The mitochondrial (mt) genomes of the dagger and needle nematodes, *Xiphinema rivesi, Xiphinema pachtaicum, Longidorus vineacola* and *Paralongidorus litoralis* were sequenced in this study. The four circular mt genomes have an estimated size of 12.6, 12.5, 13.5 and 12.7 kb, respectively. Up to date, the mt genome of *X. pachtaicum* is the smallest genome found in Nematoda. The four mt genomes contain 12 protein-coding genes (*viz. cox1-3, nad1-6, nad4L, atp6* and *cob*) and two ribosomal RNA genes (*rrnL* and *rrnS*), but the *atp8* gene was not detected. These mt genomes showed a gene arrangement very different within the Longidoridae species sequenced, with the exception of very closely related species (*X. americanum* and *X. rivesi*). The sizes of non-coding regions in the Longidoridae nematodes were very small and were present in a few places in the mt genome. Phylogenetic analysis of all coding genes showed a closer relationship between *Longidorus* and *Paralongidorus* and different phylogenetic possibilities for the three *Xiphinema* species.

The phylum Nematoda is one of the largest and most diverse groups of animal organisms, with a global distribution. Most species are found in oceanic, freshwater and soil ecosystems, and only a small number are pathogens of animals or plants. They cause reductions in agricultural productivity and disease in humans, and animals^1^. Plant-parasitic nematodes (PPNs) are distributed between Classes Chromadorea and Enoplea in only three orders *viz*. Rhabditida, Dorylaimida and Triplonchida^2^. The order Dorylaimida within Enoplea includes several genera of dagger and needle nematodes belonging to the family Longidoridae (*viz. Australodorus, Longidoroides, Longidorus, Paralongidorus, Paraxiphidorus, Xiphidorus* and *Xiphinema*)^2^. Longidoridae nematodes are highly polyphagous on wild and cultivated plants, and some are plant-virus vectors (nepovirus)^3^. Also some of the Longidoridae species are listed as A1 and A2 quarantine pests by the European and Mediterranean Plant Protection Organisation (EPPO, www.eppo.int/QUARANTINE/).

Mitochondrial (mt) genomes and sequences of individual mt genes are used to infer phylogenetic relationships among species at different taxonomic levels^4^,^5^,^6^,^7^. Animal mtDNAs are relatively constant in gene content and order, maternally inherited, and have a reduced recombination rate and high evolutionary rate^8^. There are fourteen mt genomes of PPNs sequenced to date, and only one of them was included in the class Enoplea (*Xiphinema americanum*) and fourteen in the class Chromadorea (*Aphelenchoides besseyi, Bursaphelenchus mucronatus, B. xylophilus, Globodera ellingtonae, G. pallida, G. rostochiensis, Heterodera glycines, Pratylenchus vulnus, Meloidogyne chitwoodi, M. floridensis, M. graminicola, M. hapla, M. incognita, Radopholus similis*) and some of them display some unusual features^9^,^10^,^11^,^12^,^13^,^14^,^15^,^16^,^17^,^18^,^19^,^20^,^21^. *Xiphinema americanum* mt genome encodes genes in both strands and has short coding regions^18^. On the other hand, within PPN Chromadorean species, *Globodera ellingtonae, G. pallida* and *G. rostochiensis* have multipartite mt genomes^17^,^19^,^20^, *Meloidogyne* spp. and *Pratylenchus vulnus* have a large non-coding region with tandem repeats and the control region^9^,^10^,^11^,^12^,^13^,^14^,^21^ and *Radopholus similis* has a unique genetic code, and uses the UAA codon for the aminoacid Tyr (Y) instead of a termination site^16^. Thus, there is a lack of information concerning the mt genomes for other genera within Longidoridae which is needed to derive insights into their taxonomy, phylogeny and possible molecular markers.

The main objectives of this research were: (*i*) to determine the mitochondrial genomes in three different genera within important PPNs from Longidoridae (*Xiphinema, Longidorus* and *Paralongidorus*); and (*ii*) to associate their gene structures and protein-coding gene sequences with their phylogeny.

## Results and Discussion

### General features of the mitochondrial genomes of *Longidorus vineacola, Paralongidorus litoralis, Xiphinema pachtaicum* and *Xiphinema rivesi*

**Figure 1:**
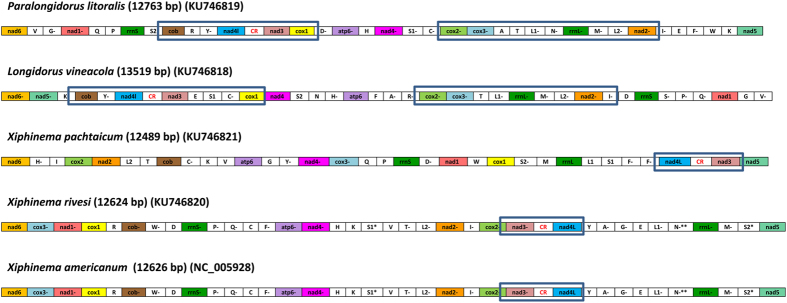
Linear maps of the mitochondrial genomes of five Longidoridae species. Positive or negative after the gene name abbreviation depends on the sense in the genome. Protein and rRNA genes have standard nomenclature. tRNA genes were designated by single-letter abbreviations. Two tRNAs for Leucine (L) and Serine (S) are present. Non-coding regions, with the exception of possible replication control region (CR), were not included.

A summary of the mt genomes in Longidoridae is shown in Table 1. The complete mt genomes of *P. litoralis, L. vineacola, X. pachtaicum* and *X. rivesi* were 12,763 bp (KU746819), 13,519 bp (KU746818), 12,489 bp (KU746821) and 12,624 bp (KU746820) in size, respectively (Fig. 1 and Table 1). The four new mt genomes showed a similar size and gene number complement to *X. americanum* (NC_005928) (12,626 bp). They are smaller in size than other Enopleans species such as *Romanomermis culicivorax* (26,194 bp) or *Hexamermis agrotis* (24,606 bp). The mt genome of *X. pachtaicum* represents the smallest mt Nematoda genome known so far (search done in GenBank, November 9, 2016).

The nucleotide composition of the mtDNA genomes studied showed an A + T content similar among dagger nematode species (66.50%, 68.86% and 68.50% for *X. americanum, X. rivesi* and *X. pachtaicum*, respectively), but slightly lower in needle nematode species (*L. vineacola* and *P. litoralis*, 63.64% and 63.89%, respectively) (Table 1). These levels were lower than that for other members of the class Enoplea such as *Romanomermis culicivorax* (79.34%) or *Hexamermis agrotis* (78.42%). The GT-rich sequences in *Xiphinema* species were 54.00% and 56.63% (G + T) in one of the strands for *X. rivesi* (GT rich strand not containing the *cox1* gene) and *X. pachtaicum* (strand containing the *cox1* gene), respectively; while, for *Longidorus* and *Paralongidorus* these differences were minimal (50.51% and 50.75%, respectively). These differences influence the coding genes, rRNA and tRNA distribution in the genome. In *X. rivesi*, the GT rich strand had sense sequences of 8 protein coding genes (PCGs), 12 tRNAs and the 2 rRNA genes whereas for *X. pachtaicum* 10 PCGs, 12 tRNAs and the 2 rRNA genes were detected. These differences were minimal for *Longidorus* and *Paralongidorus* between AC-rich *vs* GT-rich strands with 6 *vs* 6 PCGs, 10 *vs* 11 tRNAs and 1 rRNA gene in each of the strands in the case of *P. litoralis* (GT-rich strand containing the *cox1* gene) and 5 *vs* 7 PCGs proteins, 14 *vs* 8 tRNAs, and the 2 *vs* 0 rRNA genes in the case of *L. vineacola* (GT-rich strand containing the *cox1* gene).

The mtDNA genomes of *L. vineacola, P. litoralis, X. pachtaicum* and *X. rivesi* contained 12 PCGs, *viz. cox1-3, nad1-6, nad4L, atp6* and *cob*; and two ribosomal RNA genes (*rrnL* and *rrnS*) but the *atp8* gene was not detected (Fig. 1; Table 2). The gene arrangement within Longidoridae was very different within dagger and needle nematode species (Fig. 1), with the exception of *X. americanum* and *X. rivesi*, in which it was identical. This is in concordance with the very high degree of variation in mtDNA genome gene arrangements across the Metazoa^22^. A comparison of closely related species with different gene orders suggests that there are several types of “elementary” rearrangement events^5^: inversions, transpositions, inverse transpositions (i.e. a transposition in which the re-inserted fragment is inverted), and tandem duplications followed by the random loss of one of the copied genes.

### Protein encoding genes and codon usage

Protein encoding genes were transcribed from both strands in the four mt genomes sequenced. Genes *nad4L* and *nad3* were always together and separated by a non-coding region in all the studied species (Fig. 1). The gene order of two genes in *nad5-nad6, atp6-nad4* and *nad1-cox1* were conserved between *X. americanum/X. rivesi vs X. pachtaicum*. On the other hand, the gene order between *Longidorus* and *Paralongidorus* species was kept in two regions: *cob-nad4L-nad3-cox1* and *cox2-cox3-nad2*. Only the gene associations of *nad5-nad6* (inverted gene sense in *L. vineacola*) and *atp6-nad4* were kept between *Paralongidorus* and *Xiphinema* species. We could not find coincidences for arrangement of PCGs between the genus *Longidorus* and *Xiphinema*. For instance, the association between *nad5-nad6* and *atp6-nad4* seem derived from an inversion between *Longidorus* and *Paralongidorus*.

All PCGs shared an ATA start codon. The PCGs all terminated with a potential TAA stop codon with the exception of *cox3* and *nad3* for *X. rivesi*; *cox1, cox3, nad4, nad4L, nad6* and *cob* for *X. pachtaicum*, and *nad1* and *nad3* for *P. litoralis* (Table 2). Some genes in all the nematode species studied partially overlapped and probably terminated with T/TA (Table 2). These features were not conserved in these genes among the studied species. Only the *cox2* termination codon was conserved in *X. americanum, X. rivesi, X. pachtaicum* and *L. vineacola*. However, they were partially conserved between *X. americanum* and *X. rivesi* for *cox1* and *atp6* genes. Additionally to these termination codons, some genes overlap several tRNA codons and a few bases (1 or 2) with other genes (Table 2). Coding gene overlapping seems to be a common feature in Longidoridae, since the five sequenced species showed this feature in their genomes. Overlaps were detected in the same or in the opposite direction. However, gene overlap in the same sense strand was not detected in *P. litoralis*. In *X. americanum, X. rivesi* and *X. pachtaicum* a 1 bp overlap was detected between *nad2* and *cox2* in the same sense strand, *X. pachtaicum* has a 4 bp overlap between *nad4* and *cox3*, and *L. vineacola* has a 1 bp overlap between *cox2* and *cox3* in the same sense strand in both cases. In the case of overlapping coding genes in humans (*atp8/atp6* and *nad4L/nad4*) both pairs of genes result in dicistronic messengers^23^. In Ascidian species, two cases of overlapped genes, expressed as transcripts with no poly(A) tails downstream of the first open reading frame, suggested the presence of mature bicistronic transcripts^24^.

The majority of genes kept the transcription sense with 2 or more consecutive genes, but there are exceptions where unique genes have a different sense to other genes in the mitochondria, as *cox1* in the case of *X. americanum* and *X. rivesi*, and *nad1* in the case of *L. vineacola* and *P. litoralis*. It is suggested that gene arrangement is affected by the transcription mechanism, for example by the need to co-regulate the expression of some genes or the required stoichiometry of the gene products^22^.

As in other published nematode mtDNAs, the mtPCGs of our sequenced species were notably biased toward using amino acids encoded by T-rich codons^13^ (Table 3). The three most frequently used codons were TTT, TTA and ATT (Table 3) in *Xiphinema* species. These T-rich codons accounted for 21.97% (*X. americanum*), 24.37% (*X. rivesi*) and 23.68% (*X. pachtaicum*) of the total codons used. In the case of *Longidorus* and *Paralongidorus* the most used triplets were TTA, TTT and ATA. These codons account for 18.61% (*P. litoralis*) and 17.01% (*L. vineacola*) of the total codons used. However, these differences in T-rich codons were smaller in comparison to other Chromadorean nematodes such as *B. xylophilus* or *P. vulnus*, which account for 40.30% and 31.44% of the total codons used, respectively, while our range of the most used codons was similar to other Enoplean nematodes such as *Trichinella* or *Trichuris* species^25^,^26^. The higher frequency of amino acids encoded by T-rich codons, and unequal synonymous codon usage with bias against C-rich codons is consistent with the high percentage of A + T content in the nucleotide composition of PCGs as in other mt genomes for nematodes^13^.

### Transfer RNA (tRNA) and ribosomal RNA genes

The mt genome of metazoans typically encodes 22 tRNAs^8^. However, only *L. vineacola* showed the typical 22 tRNAs, whereas 21 tRNAs were identified in *X. rivesi* and in *P. litoralis* in which we could not identify the mt-tRNA^N^ gene, and 19 tRNAs were identified in *X. pachtaicum* in which we could not identify the mt-tRNA^N^, mt-tRNA^A^ and mt-tRNA^R^ genes. The position predicted for mt-tRNA^N^ in mt genomes of *X. rivesi* and *P. litoralis* based on sequence similarity in the genes predicted by Jühling *et al*. was deep inside the l-rRNA annotation and in the same sense strand^27^. The annotation of our l-rRNA was based on similarity to sequences deposited in GenBank and mainly with the *X. americanum* l-rRNA annotation done by He *et al*. using mRNA sequencing^18^. The same situation was also found in *X. pachtaicum* for mt-tRNA^N^ where additionally, the other two mt-RNAs, mt-tRNA^A^ and mt-tRNA^R^, were not found. These tRNAs would therefore need to be imported from the nucleus, implying that a mechanism of this sort exists in nematodes. Import of tRNA from the nucleus to the mitochondrion has been demonstrated in marsupials^28^ and in a protozoan (*Trypanosoma bruceii*)^29^. Another possibility is that the tRNA detection methods used in this study (Mitos-MITFI and Arwen v1.2) could not identify them.

The tRNA structures detected in the four studied mtDNA genomes shared some features with those of other metazoans^30^ including a 5 bp anticodon stem, a 7 base anticodon loop with a T always preceding an anticodon as well as a purine always following an anticodon (Figs S1–S3). Four secondary structures have been found in Longidoridae (Table 4; Figs S1–S3): (i) typical nematode tRNAs structure with a D-arm but no T-arm; (ii) structure retaining their T-arm and lacking the D-arm; (iii) structures lacking both the T-arm and the D-arm; and (iv) the intact clover-leaf structure is also present. The conventional cloverleaf structure it is also present in the mt-tRNAs of *Trichuris ovis, Trichuris discolor*^26^ and *Trichinella spiralis*^31^. All of the tRNAs with a clover-leaf structure found in the species included in this study were coincident with those found in *T. spiralis* and only the tRNA^Y^ which showed a clover-leaf structure in *X. rivesi*, does not appear with this secondary structure in *T. spiralis*. These diversity of structures in our mt tRNAs support the hypothesis expressed by Jühling *et al*., that mt-tRNA not only evolve rapidly at the sequence level but also exhibit a variety of deviations from the common clover-leaf structure^27^.

### Non-coding regions

**Figure 2:**
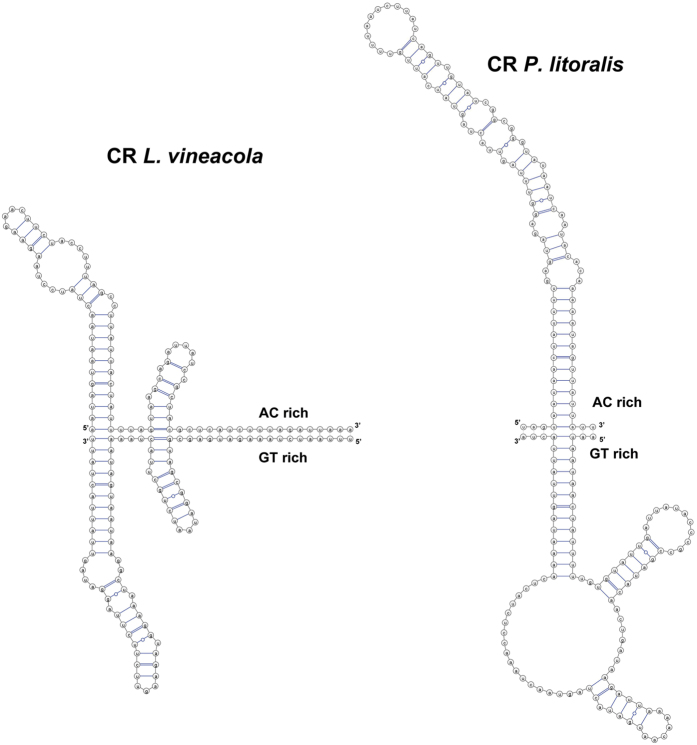
Stem-loop structures predicted for the noncoding region between the *nad4L* and *nad3* genes (replication control region, CR) for *Longidorus vineacola* and *Paralongidorus litoralis*.

The sizes of the non-coding regions in Longidoridae nematodes in this study were very small and in few places in the mt genome (Table 5). In the mt genome of *X. americanum*, the longest noncoding region was just 96 bp in length between the *nad3* and the *nad4L* genes with an A + T content of 72%, and inverted or direct repeats were not present^32^. A sequence motif (5′-GAGACCTGAGCCCAAGATA-3′) was present in this 96-bp noncoding region for *X. americanum*^32^ that was similar to the conserved promoter element sequence (5-CA(G)ACC(G)CC(A)AAAGATA-3) around the transcription start site in the D-loop region of the human mt genome^33^. We could not find a clear similarity in our non-coding sequences to this promoter element sequence. However, the position of a non-coding region and a stable gene arrangement between *nad3* and *nad4l* within all the studied Longidoridae nematodes pointed out the importance of this region in the viability of the nematode mitochondria. This feature, the strong secondary structure (Figs 2 and S4) and the location of the Control Region (CR) for *X. americanum* were the basis for annotating this sequence as CR. The CRs were of different size and composition in comparison to the A + T rich sequence found in *X. americanum*. Sizes of 93, 103, 96, 110, and 140 bps with A + T contents of 70%, 74%,72%, 73% and 75% were found in the case of *L. vineacola, P. litoralis, X. americanum, X. rivesi* and *X. pachtaicum*, respectively. For *X. americanum, X. rivesi* and *X. pachtaicum*, the A quantity was higher than T (*X. americanum* = A: 55.21% *vs* T: 16.67%; *X. rivesi* = A: 55.21% *vs* T: 17.71%; *X. pachtaicum* = A: 50.71% *vs* T: 24.29%), while for *L. vineacola* and *P. litoralis* it was more balanced between A and T (*L. vineacola* = A: 34.41% *vs* T: 35.48%; *P. litoralis* = A: 33.01% *vs* T: 40.78%). We could not find a good explanation for these ratios, but in the case of *P. litoralis* and *L. vineacola*, this region was accompanied with strong secondary structures and a similar number of coding genes in both strands. Probably, this composition has strong effects on replication and transcription. Lewis *et al*. found that mtDNA synthesis in the *C. elegans* gonad produces branched-circular lariat structures with multimeric DNA tails; they were able to detect multimers up to four mtDNA genome unit lengths^34^. These authors have raised the possibility that the rolling circle mtDNA replication mechanism may be an ancestral trait among metazoans. However, *C. elegans* has two well-defined non-coding regions and with our data only one was detected and in a similar position in the studied Longidoridae species. Many species of Arthropoda, Nematoda, Mollusca and Annelida harbor palindromes and inverted repeats in their CRs^35^. Length of the CR in the nematode mtDNA genome could be variable because of the presence of repeated sequences^22^. We only found a partially conserved palindrome sequence in the CR of *P. litoralis* (5′-TAGTAATAACTATTTTCAGTA-3′) applying the procedure explained in Arunkumar and Nagaraju^35^.

Another interesting aspect of these mt genomes was in the number of short non-coding regions and in their distribution in different positions in the genome ranged from 1 to 6. We found important differences between the studied species (excluding CR) (Table 5). The number of non-coding regions longer than 30 bp ranged from 1 to 6 non-coding regions. Non-coding regions longer than 50 bp were only found in *L. vineacola* and *P. litoralis* (4 and 1, respectively; Table 5). Secondary structures were drawn in Figs S5 and S6. Long non-coding regions showed strong structures, but they were located in different regions in *L. vineacola* and *P. litoralis*. Some non-coding regions were at the same position in all the studied species, as it is the case between tRNA^N^ and tRNA^Q^, with a similar size (from 36 to 42 bp). This is coincident with the non-coding region having a strong arm structure (Figs S5 and S6). It is possible that these regions function as splicing recognition sites during processing of the transcripts. Mitochondrial genomes in Longidoridae have the important trait of genome size economy and in having fewer non-coding regions. These features were retained even with the important differences in gene arrangements between the studied sequences.

### Mitochondrial phylogeny of Enoplea nematodes

**Figure 3:**
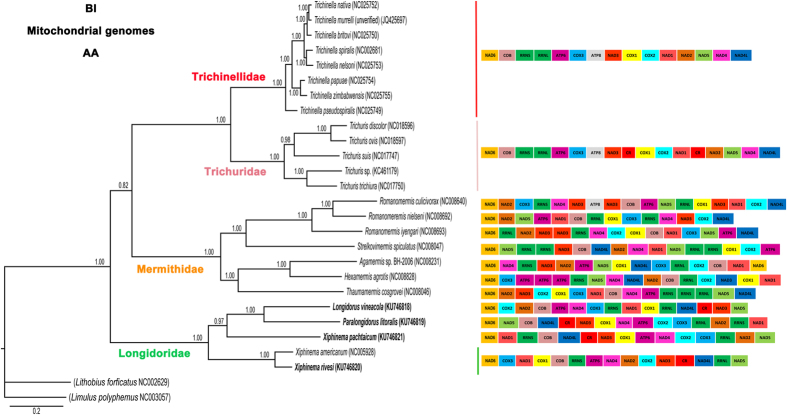
Inferred phylogenetic tree resulting from Bayesian analysis of amino acid dataset for 13 protein-coding genes from Enoplea and two arthropod outgroups. Bayesian probability values (BPP) shown above the node (≥70).

**Figure 4:**
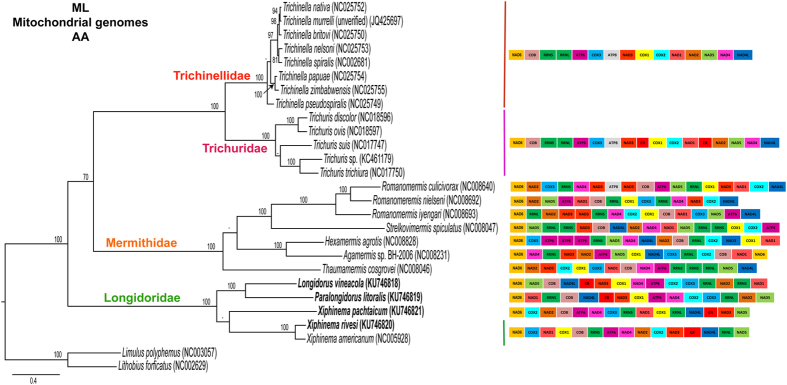
Single maximum likelihood tree with values from the separate bootstrap analysis and two arthropod outgroups. Analysis of amino acid sequences for 13 protein-coding genes inferred using RAxML (see methods for analysis details). Bootstrap values shown above the node (≥70).

We conducted BI and ML phylogenetic analyses of an amino acid sequence dataset (13 protein-coding genes) for 25 nematode species. Phylogenetic analysis separated four different clades and subclades including Trichinellidae, Trichuridae, Mermithidae and Longidoridae (Figs 3 and 4). In our analysis, Trichinellidae and Trichuridae formed a well-supported group which is sister to Mermithidae, and all these three groups formed a clade weakly supported in both analysis (BI and ML). This phylogenetic position is mainly consistent with recent reports based on mt genome analysis^11^,^36^, but those were only based on one sequence for Longidoridae (*viz. X. americanum*). Meanwhile, Longidoridae clade, with 5 representatives in our analysis, was a sister clade with the other groups in the Bayesian inference (BI) and Maximum likelihood (ML) analyses in the amino acid dataset (Figs 3 and 4). In Longidoridae, two well-supported subclades were formed, one of them with *Longidorus, Paralongidorus* and *X. pachtaicum* with Bayesian probability values (BPP) of 0.97 in the BI analysis, while the position of *X. pachtaicum* was not associated to any specific clade in the ML analysis, and another with the other *Xiphinema* species. *Xiphinema americanum* and *X. rivesi* were closely related phylogenetically and this is shown with the strong support of their relationship in both analyses (100% bootstrap support (BS) and 1.00 BPP), while the position of *X. pachtaicum* was well resolved in this clade by BI (1.00 BPP), but not by ML (65% BS). Nucleotide phylogenetic analysis showed a similar pattern of well-supported phylogenetic clades as in the aminoacid dataset forming two sister clades, one with Trichinellidae and Trichuridae and another with Mermithidae and Longidoridae in BI and ML analysis (Figs S7 and S8). However, this phylogenetic position with Longidoridae as a sister clade to Mermithidae was well-supported in BI and weakly supported in ML analysis. Longidoridae clade was formed by two subclades, one for *Paralongidorus* and *Longidorus* and the other for *Xiphinema* species. This clade was strongly supported in the BI analysis and weakly supported in the ML analysis. *Xiphinema americanum* and *X. rivesi* were closely related phylogenetically as in the case of amino acid dataset (100% BS and 1.00 BPP). This different position of the Longidoridae clade was shown by other researchers with only one sequence from Longidoridae (*X. americanum*)^11^,^36^. The results of Kim *et al*.^36^ were similar to our phylogenetic analysis using a BI approach and using amino acid and nucleotides datasets, while the phylogenetic approach used by Humphreys-Pereira and Elling^11^ was similar in the amino acid dataset and different in the nucleotide dataset excluding the third codon position. In order to assess whether the phylogenetic relationships recovered by the mt genome sequences were influenced by the use of an entirely maternal marker, we assessed the phylogeny of the Enoplea using an available nuclear marker, partial 18S rRNA. Enoplea partial 18S (Fig. S9) showed two main well-supported clades (100% BPP) for Enoplia and Dorylaimia. Longidoridae species were closely related phylogenetically and forming a well-supported clade (100% BPP) with Nordiidae, Qudsianematidae, Dorylaimidae and Aporcelaimidae. However, no member of these families has a complete mt genome currently available. The Longidoridae clade was formed by two subclades, the superior subclade is well-supported (BPP = 0.99) comprising four different genera, *Longidorus, Paralongidorus, Xiphinema americanum* group and *Xiphidorus. Longidorus* species were phylogenetically related with *Paralongidorus* species forming a well-supported clade (BPP = 1.00) and the *Xiphinema americanum* group which formed a sister-clade with *Xiphidorus*, however the BPP values for this sister-clade is moderately supported (BPP = 0.95). The second subclade is well-supported (BI = 1.00) and was formed by *Xiphinema* non-*americanum* group species. The relationships between these three groups were not well-defined in these analyses or in other phylogenetic analysis using other phylogenetic markers with more Longidoridae species^37^. Unfortunately, we could not obtain a complete mt genome sequence for a *Xiphinema* non-*americanum* group species, so this point could not be resolved. Taking into account the sequence evolution in both clades (*Longidorus*-*Paralongidorus*) and (*Xiphinema*-*Xiphidorus*) neither of these clades seems more ancient than the others. But, clearly there are parallels in gene arrangements, phylogenetic relationships and non-coding regions between *Longidorus* and *Paralongidorus*. Additionally, *L. vineacola* has the longest non-coding regions followed by *P. litoralis*. For this reason, we hypothesized a less evolved mt genome or different selection pressure in the evolution of these species. Another observation found in recent phylogenetic analysis, is the extreme diversity of some regions (*cox1* gene) with *Longidorus orientalis* showing incongruence of phylogenies inferred from ITS1 rRNA and *cox1* genes^38^ while other species show low differences within the populations sampled (i.e. *X. pachtaicum* and *X. index*)^39^. The high variation observed in the *cox1* priming sites in *L. orientalis* can adversely affect the certainty of the nematode identification by barcoding^38^ and thus integrative taxonomical approaches are needed for an accurate identification of these and other plant-parasitic nematodes^40^,^41^,^42^. Our complete mt sequences for *Xiphinema, Longidorus* and *Paralongidorus* genera could help in resolving these problems by comparing sequences for primer design.

While mapping the gene arrangement with the species in this study, we found an important variability in Enoplea (Figs 3 and 4). Some genera such as *Trichinella* and *Trichuris* were homogenous with their PCG arrangements, however, the genus *Romanomermis* showed an important variability in its arrangement in a similar way to Longidoridae species. We could not find similarities in the PCG arrangement between the species studied, with the exception of very closely related species (*X. americanum* and *X. rivesi*).

## Material and Methods

### Samples and nematode extraction

Soil samples from which nematodes were extracted were collected in 2015 in Spain from several crops and wild habitats (Table 6). Soil samples were collected with a shovel discarding the upper 5-cm topsoil profile, from a depth of 5- to 40-cm, in the close vicinity of active roots. Nematodes from the soil were extracted from a 500-cm^3^ sub-sample using the magnesium sulphate centrifugal-flotation method^43^. *Xiphinema pachtaicum, X. rivesi, L. vineacola*, and *P. litoralis* were identified using integrative taxonomy as described in previous studies^37^,^42^,^44^,^45^. Only live and individual nematodes were used for DNA extraction. No pure populations were multiplied in pots in greenhouse and nematodes were extracted from original sampling points.

### Mitochondrial DNA extraction and amplification

For the molecular analyses, in order to avoid complications from mixed populations in the same sample, at least two live nematodes from each sample were temporarily mounted in a drop of 1 M NaCl containing glass beads (to avoid crushing the nematode) and diagnostic morphological characters were observed and measurements were taken to confirm the species identity. The slides were then dismantled and DNA extracted. Nematode DNA was extracted from single individuals and PCR assays were conducted as described by Subbotin *et al*.^46^. A portion of the *cox1* gene was amplified as described by Lazarova *et al*. using primers COIF (5′-GATTTTTTGGKCATCCWGARG-3′) and COIR (5′-CWACATAATAAGTATCATG-3′)^47^ and PCR cycling conditions as described by He *et al*.^32^. PCR products were purified using ExoSAP-IT (Affmetrix, USB products) and used for direct sequencing in both directions. The resulting products were run on a DNA multicapillary sequencer (Model 3130XL genetic analyzer; Applied Biosystems, Foster City, CA, USA), using the BigDye Terminator Sequencing Kit v.3.1 (Applied Biosystems, Foster City, CA, USA), at the Stab Vida sequencing facilities (Caparica, Portugal).

For all mt DNA amplification, the DNA extraction protocol was similar to that described in Subbotin *et al*.^38^ with the exception that several live nematodes previously identified under microscope were used for each extraction and the proteinase K digestion was performed at 50 °C. Primers were designed using the *cox1* sequences for each species. The primer design was performed using Primer3^48^ (Table 6) with the correct sense for long-range PCR which was carried out using Advantage® 2 PCR Kit (Clontech, Takara Biotechnology, Japan). Each reaction contained 0.3 μM primer, 1X BD Advantage 2 PCR buffer and 2 μl of DNA nematode extraction in a final PCR volume of 25 μl. Long-range PCR conditions were as follows: initial denaturation at 94 °C for 3 min followed by 40 cycles of 94 °C for 15 s, annealing between 55 and 57 °C depending on the primer (Table 6) for 30 s and extension at 68 °C for 15 min during the first 10 cycles and after 15 min + 15 s/cycle. The product was visualized using an agarose gel; gel purified using Cut & Spin (Grisp, Portugal) and quantified using a Nanodrop spectrophotometer.

### Ion-torrent Sequencing and read processing

Ion-torrent sequencing platform was performed at Stab Vida sequencing facilities (Caparica, Portugal). The 200–300 bp insert size library was constructed using enzymatic fragmentation of amplified DNA, Ion-torrent specific adapter ligation, size selection and amplification. The concentration and size distribution of library DNA fragments was determined using Qubit® fluorometer 2.0 (Invitrogen) and Bioanalyzer 2100 (Agilent Technologies). The numbers of reads obtained were 351,698; 665,110; 339,777; and 596,021 for *X. rivesi, X. pachtaicum, L. vineacola* and *P. litoralis*, respectively. Raw data obtained were analyzed and trimmed using CLC Genomics Workbench 7.5.1 (Qiagen) following standard procedures described by the manufacturer in Stab Vida facilities.

### Mitochondrial genome assembly and annotation

Filtered data was *de novo* assembled using CLC Genomics Workbench 7.5.1 (Qiagen). Prediction of protein-coding genes and rRNA genes was done by using a combination of BLAST searches in Artemis v. 16.0.0^49^, and MITOS online software^50^. Putative tRNA (transfer RNA) genes were identified in the MITOS online software^50^, which uses the strategy presented in Jühling *et al*.^27^. Additionally, these tRNA predictions were checked using the program Arwen v1.2^51^. The assembled genomes were annotated using Artemis v. 16.0.0^49^. Annotated sequences were submitted to GenBank with the accession numbers KU746820, KU746821, KU746818 and KU746819 for *X. rivesi, X. pachtaicum, L. vineacola* and *P. litoralis*, respectively. Codon usage was studied on-line using the server http://www.bioinformatics.org/sms2/codon_usage.html.

### Phylogenetic analyses

Phylogenetic analyses were performed on amino acid (AA) and nucleotide data sets and the nuclear partial 18S rRNA. The newly obtained and published sequences for mt coding genes in complete Enoplea mt genomes were used for phylogenetic reconstruction. *Lithobius forficatus* (NC002629) and *Limulus polyphemus* (NC003057) were used as outgroups, according to previous studies^11^,^36^. For multiple alignments of AA sequences, the nucleotide sequences of each of the protein coding genes (PCG) were initially translated into AA with MEGA6^52^ using the invertebrate mt genetic code setting. The amino acid sequences for each PCG were aligned individually using ClustalW^53^, implemented in MEGA6 under default settings. Conserved regions in the alignments of the 13 PCGs were selected using the Gblocks v 0.91b server set at the “less stringent” option (allow smaller final blocks, allow gap positions within the final blocks and allow less strict flanking positions)^54^. Each individual gene alignment was tested for the best-fit substitution model using ProtTest 2.4^55^ based on the Akaike information criterion (AIC). All AA alignments of the 13 PCGs were concatenated into a single alignment using Mesquite v3.04^56^. The final alignment included 2468 out of 3926 AA, representing 63% of the original sequence alignment. Similarly, aligned nucleotide sequences from the PCG using the aligned amino acid sequences were trimmed, concatenated in a similar procedure as for AA dataset. The best fitted model of DNA evolution for each individual gene alignment was obtained using jModelTest v. 2.1.7^57^ with AIC. Model used in both datasets are shown in Table S1. Selected models from the available programs in MrBayes 3.1.2 and RAxML 8.2.2 which best fit our dataset from the ranked models in Protest 3.2 and jModelTest 2.1.7. were used in the phylogenetic analysis

The partial 18S rRNA data set consisted of 88 Enoplea sequences from GenBank and was 1666 bp in length, and comprised representatives of the main families. *Lithobius forficatus* (EU024571) and *Limulus polyphemus* (HQ588741). Sequences for partial 18S were aligned using MAFFT v. 7.205^58^. Conserved regions in the alignments were trimmed using the Gblocks v 0.91b server set at the “less stringent” option^54^. The best fitted model of DNA evolution was obtained using jModelTest v. 2.1.7^57^ with AIC.

Phylogenetic analyses of the AA data sets were performed based on maximum likelihood using the rapid bootstrap algorithm in RAxML v. 8.2.2^59^ with 200 bootstrap replicates. BI was performed using MrBayes 3.1.2^60^. In both cases, the models were included in the analysis. BI was performed including the model in a partition setting with the model for each PCG and for nucleotides and was run under a general time reversible of invariable sites and a gamma-shaped distribution (GTR + I + G) model with four chains for 1 × 10^6^ generations. After discarding burn-in samples and evaluating convergence, the remaining samples were retained for further analyses. The topologies were used to generate a 50% majority rule consensus tree. Trees were visualized using TreeView^61^ and FigTree v1.4.2 (http://tree.bio.ed.ac.uk/software/figtree/).

## Additional Information

**How to cite this article**: Palomares-Rius, J. E. *et al*. Mitochondrial genome diversity in dagger and needle nematodes (Nematoda: Longidoridae). *Sci. Rep.* **7**, 41813; doi: 10.1038/srep41813 (2017).

**Publisher's note:** Springer Nature remains neutral with regard to jurisdictional claims in published maps and institutional affiliations.

## Supplementary Material

Supplementary Information

## Figures and Tables

**Table 1:** Nucleotide composition of mitochondrial genomes within Longidoridae.

Nucleotide	*Xiphinema americanum* (NC_0055928)	*Xiphinema pachtaicum* (KU746821)	*Xiphinema rivesi* (KU746820)	*Longidorus vineacola* (KU746818)	*Paralongidorus litoralis* (KU746819)
**Length (bp)**					
Entire Sequence	12,626	12,489	12,624	13,519	12,763
Protein coding sequence	10,014	9,921	10,116	10,099	10,002
Ribosomal RNA gene sequence	1,298	1,238	1,295	1,272	1,326
Transfer RNA gene sequence	1,012	1,051	1,111	1,214	1,159
Non coding region	166	397	161	973	371
**A** **+** **T (%)**					
Entire Sequence	66.50	68.50	68.86	63.64	63.89
Protein coding sequence	66.67	68.45	69.41	63.53	63.89
**Codon position**					
1^st^ codon	64.15	65.59	65.77	59.64	60.54
2^nd^ codon	64.44	65.35	64.97	61.54	63.23
3^rd^ codon	71.14	74.30	77.26	69.11	67.61

**Table 2:** Comparison of mitochondrial and rRNA genes within Longidoridae.

Protein genes	*Xiphinema americanum*			*Xiphinema rivesi*			*Xiphinema pachtaicum*			*Paralongidorus litoralis*			*Longidorus vineacola*		
	AA	Predicted initiation and termination codons		AA	Predicted initiation and termination codons		AA	Predicted initiation and termination codons		AA	Predicted initiation and termination codons		AA	Predicted initiation and termination codons	
*cox1*	518	ATA**,^b^	T(AA)	515	ATA**,^b^	T(AA)*,^a^	513	ATA*,^a^	TAG	512	ATA	TAA	515	ATA	TAA*,^b^
*cox2*	206	ATA	TA(A)**,^a^	206	ATA	TA(A)**,^a^	186	ATA	TA(A)**,^a^	207	ATA	TAA*,^a^	200	ATA**,^a^	TA(A)*,^a^
*cox3*	249	ATA	TAA**,^b^	249	ATA	TAG**,^b^	256	ATA	TAG**,^a^	241	ATA	TAA	258	ATA	TA(A)**,^a^
*nad1*	198	ATA**,^b^	TAA	289	ATA**,^b^	TAA	276	ATA	TA(A)*,^a^	305	ATA	TAG*,^a^	292	ATA	T(AA)*,^a^
*nad2*	278	ATA**,^a^	T	280	ATA**,^a^	TAA*,^a^	280	ATA**,^a^	TAA*,^a^	280	ATA	TAA	273	ATA	TAA*,^a^
*nad3*	106	ATA	T(AG)	106	ATA	TAG*,^a^	112	ATA	TAA	109	ATA	TAG	111	ATA	TAA
*nad4*	391	ATA*,^a^	TAA	391	ATA	TAA	388	ATA**,^a^	TAG*,^a^	364	ATA	TAA*,^b^	387	ATA	TAA
*nad4L*	91	ATA	TAA	89	ATA*,^b^	TAA	73	ATA	TAG	90	ATA*,^b^	TAA	84	ATA	TAA
*nad5*	515	ATA	TAA	518	ATA*,^a^	TAA	497	ATA	TAA^b^	504	ATA	TAA	512	ATA*,^a^	TAA
*nad6*	146	ATA	TAA**,^b^	146	ATA	TAA**,^b^	144	ATA	TAG*,^b^	142	ATA	TAA	146	ATA	TA(A)*,^a^
*cob*	366	ATA	TAA*,^a^	366	ATA	TAA*,^b^	367	ATA	TAG*,^b^	365	ATA	T(AA)*,^a^	366	ATA	TAA
*atp6*	205	ATA	T(AA)	205	ATA	TA(A)*,^a^	203	ATA	TAA*,^a^	203	ATA*,^b^	TAA	205	ATA*,^b^	TAA
*atp8*	NF	NA	NA	NF	NA	NA	NF	NA	NA	NF	NA	NA	NF	NA	NA
**rRNA genes**															
*rrnL*	729				724		707			720			722		
*rrnS*	569				571		531			606			546		

^*^Partially overlap a tRNA.

^**^Partially overlap a gene.

^a^In same strand sense overlapping.

^b^In opposite strand sense overlapping.

**Table 3:** Codon usage of 12 protein coding genes of *Xiphinema americanum* (Xa), *Xiphinema rivesi* (Xr), *Xiphinema pachtaicum* (Xp), *Paralongidorus litoralis* and *Longidorus vineacola* (Lv) mtDNAs.

AA	Codon	No.*					%***					AA	Codon	No.*					%***				
		**Xa	Xr	Xp	Pl	Lv	Xa	Xr	Xp	Pl	Lv			Xa	Xr	Xp	Pl	Lv	Xa	Xr	Xp	Pl	Lv
**Ala**	**GCG**	11	9	10	19	17	0.33	0.27	0.31	0.57	0.51	**Lys**	**AAG**	32	30	31	28	22	0.95	0.89	0.95	0.85	0.66
**Ala**	**GCA**	32	33	18	29	49	0.95	0.98	0.55	0.88	1.46	**Lys**	**AAA**	46	50	61	60	62	1.37	1.49	1.87	1.81	1.85
**Ala**	**GCT**	69	78	57	61	74	2.05	2.32	1.74	1.84	2.21	**Met**	**ATG**	64	47	54	52	48	1.91	1.40	1.65	1.57	1.43
**Ala**	**GCC**	19	10	28	39	41	0.57	0.30	0.86	1.18	1.22	**Met**	**ATA**	130	161	151	170	156	3.87	4.79	4.62	5.14	4.65
**Arg**	**CGG**	5	6	6	7	8	0.15	0.18	0.18	0.21	0.24	**Phe**	**TTT**	297	312	315	199	149	8.84	9.29	9.64	6.01	4.44
**Arg**	**CGA**	18	21	16	9	21	0.54	0.63	0.49	0.27	0.63	**Phe**	**TTC**	82	78	50	65	99	2.44	2.32	1.53	1.96	2.95
**Arg**	**CGT**	12	11	18	9	4	0.36	0.33	0.55	0.27	0.12	**Pro**	**CCG**	22	16	7	7	7	0.66	0.48	0.21	0.21	0.21
**Arg**	**CGC**	5	2	3	10	2	0.15	0.06	0.09	0.30	0.06	**Pro**	**CCA**	29	37	29	21	33	0.86	1.10	0.89	0.63	0.98
**Asn**	**AAT**	62	71	63	50	42	1.85	2.11	1.93	1.51	1.25	**Pro**	**CCT**	48	49	43	59	46	1.43	1.46	1.32	1.78	1.37
**Asn**	**AAC**	29	21	25	45	40	0.86	0.63	0.76	1.36	1.19	**Pro**	**CCC**	14	9	16	20	23	0.42	0.27	0.49	0.60	0.69
**Asp**	**GAT**	35	38	34	24	26	1.04	1.13	1.04	0.73	0.77	**Ser**	**AGG**	56	42	36	65	50	1.67	1.25	1.10	1.96	1.49
**Asp**	**GAC**	15	9	12	26	18	0.45	0.27	0.37	0.79	0.54	**Ser**	**AGA**	78	83	93	82	113	2.32	2.47	2.84	2.48	3.37
**Cys**	**TGT**	34	35	36	23	18	1.01	1.04	1.10	0.69	0.54	**Ser**	**AGT**	46	65	68	58	56	1.37	1.93	2.08	1.75	1.67
**Cys**	**TGC**	17	7	11	9	13	0.51	0.21	0.34	0.27	0.39	**Ser**	**AGC**	19	13	16	29	33	0.57	0.39	0.49	0.88	0.98
**Gln**	**CAG**	22	11	14	18	13	0.66	0.33	0.43	0.54	0.39	**Ser**	**TCG**	25	13	20	16	9	0.74	0.39	0.61	0.48	0.27
**Gln**	**CAA**	33	44	41	33	39	0.98	1.31	1.25	1.00	1.16	**Ser**	**TCA**	55	61	38	31	40	1.64	1.82	1.16	0.94	1.19
**Glu**	**GAG**	24	28	25	31	26	0.71	0.83	0.76	0.94	0.77	**Ser**	**TCT**	97	110	95	80	92	2.89	3.27	2.91	2.42	2.74
**Glu**	**GAA**	42	42	58	40	47	1.25	1.25	1.77	1.21	1.40	**Ser**	**TCC**	42	29	30	33	38	1.25	0.86	0.92	1.00	1.13
**Gly**	**GGG**	41	37	38	81	78	1.22	1.10	1.16	2.45	2.32	**Thr**	**ACG**	17	15	17	17	15	0.51	0.45	0.52	0.51	0.45
**Gly**	**GGA**	77	90	78	78	84	2.29	2.68	2.39	2.36	2.50	**Thr**	**ACA**	30	34	39	53	50	0.89	1.01	1.19	1.60	1.49
**Gly**	**GGT**	56	48	44	35	31	1.67	1.43	1.35	1.06	0.92	**Thr**	**ACT**	72	76	68	84	85	2.14	2.26	2.08	2.54	2.53
**Gly**	**GGC**	15	14	25	18	20	0.45	0.42	0.76	0.54	0.60	**Thr**	**ACC**	27	22	25	32	41	0.80	0.65	0.76	0.97	1.22
**His**	**CAT**	38	40	39	41	38	1.13	1.19	1.19	1.24	1.13	**Trp**	**TGG**	36	28	33	38	20	1.07	0.83	1.01	1.15	0.60
**His**	**CAC**	19	19	23	26	25	0.57	0.57	0.70	0.79	0.74	**Trp**	**TGA**	70	74	72	65	80	2.08	2.20	2.20	1.96	2.38
**Ile**	**ATT**	186	203	197	136	142	5.54	6.04	6.03	4.11	4.23	**Tyr**	**TAT**	71	73	75	53	59	2.11	2.17	2.29	1.60	1.76
**Ile**	**ATC**	50	46	36	53	68	1.49	1.37	1.10	1.60	2.03	**Tyr**	**TAC**	25	18	20	43	41	0.74	0.54	0.61	1.30	1.22
**Leu**	**TTG**	104	88	116	88	54	3.10	2.62	3.55	2.66	1.61	**Val**	**GTG**	54	42	46	50	53	1.61	1.25	1.41	1.51	1.58
**Leu**	**TTA**	255	305	262	247	266	7.59	9.08	8.01	7.46	7.93	**Val**	**GTA**	95	93	83	118	136	2.83	2.77	2.54	3.56	4.05
**Leu**	**CTG**	29	19	25	53	34	0.86	0.57	0.76	1.60	1.01	**Val**	**GTT**	98	100	112	97	68	2.92	2.98	3.43	2.93	2.03
**Leu**	**CTA**	79	67	65	111	133	2.35	1.99	1.99	3.35	3.96	**Val**	**GTC**	23	18	16	29	36	0.68	0.54	0.49	0.88	1.07
**Leu**	**CTT**	99	92	75	82	80	2.95	2.74	2.29	2.48	2.38												
**Leu**	**CTC**	26	18	12	25	45	0.77	0.54	0.37	0.76	1.34												

^*^No.  = number in all coding genes.

^**^Xa = *Xiphinema americanum*; Xr = *Xiphinema rivesi*; Xp = *Xiphinema pachtaicum*; Pl = *Paralongidorus litoralis*; and Lv = *Longidorus vineacola*.

^***^% = percentage in respect to the total number of codons in the genome.

**Table 4:** Secondary structures of predicted mt-RNAs in selected Enoplea species.

Organism	A*	C	D	E	F	G	H	I	K	L1	L2	M	N	P	Q	R	S1	S2	T	V	W	Y
**Dorylaimida**																						
*Xiphinema americanum*	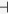	|	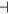	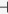	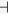	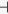	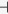	|	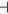	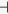	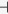	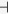	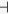	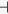	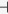	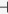	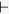	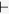	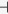	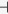	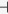	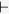
*Xiphinema rivesi*	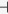	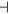	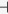	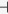	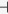	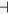	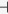	|	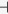	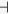	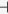	+	nf	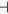	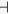	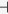	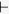	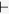	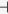	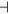	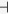	+
*Xiphinema pachtaicum*	nf	+	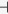	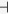	+	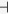	|	+	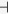	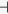	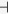	+	nf	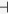	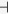	nf	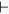	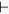	|	+	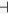	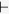
*Paralongidorus litoralis*	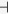	|	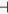	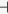	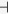	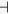	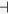	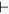	+	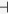	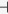	+	nf	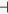	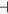	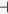	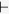	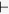	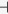	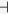	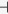	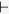
*Longidorus vineacola*	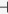	|	+	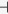	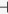	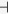	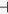	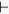	+	+	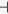	+	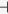	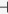	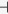	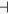	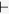	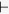	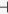	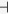	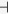	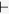
**Mermithida**																						
*Agamermis* sp BH-2006	|	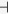	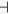	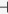	|	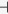	|	|	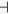	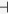	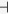	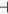	|	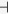	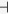	|	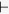	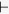	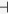		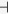	|
*Hexamermis agrotis*	|	|	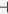	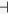	|	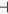	|	|	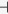	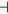	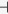	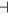	|	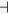	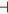	|	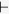	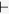	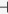	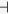	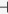	|
*Romanomermis culicivorax*	|	|	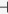	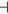	|	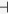	|	|	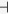	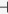	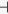	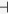	|	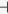	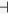	|	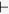	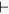	|	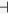	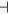	|
*Romanomermis iyengari*	|	|	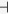	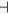	|	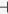	|	|	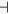	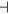	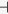	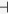	|	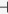	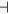	|	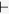	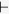	|	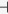	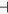	|
*Romanomermis nielseni*	|	|	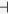	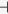	|	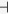	|	|	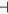	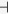	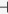	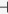	|	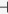	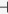	|	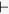	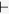	|	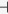	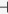	|
*Strelkovimermis spiculatus*	|	|	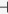	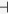	|	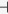	|	|	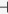	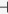	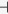	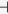	|	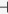	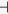	|	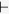	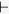	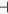	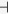	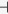	|
*Thaumamermis cosgrovei*	|	|	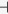	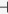	|	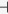	|	|	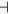	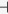	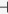	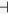	|	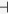	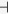	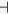	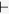	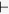	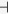	|	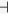	|
**Trichocephalida**																						
*Trichinella spiralis*	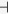	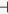	+	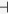	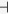	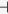	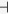	+	+	+	+	+	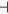	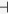	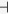	+	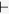	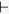	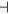	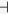	+	|

Up-dated with the species sequenced in this study from Jühling *et al*.^27^.

^*^The first row enumerates all 20 amino acids (A = alanine; C = cysteine; D = aspartic acid; E = glutamic acid; F = phenylalanine; G = glycine; H = histidine; I = isoleucine; K = lysine; L1 = leucine 1; L2 = leucine 2; M = methionine; N = asparagine; P = proline; Q = glutamine; R = arginine; S1 = serine 1; S2 = serine 2; T = threonine; V = valine; W = tryptophan; Y = tyrosine. As mitochondrial genomes encode two distinct tRNA^Leu^ and tRNA^Ser^ genes, both are listed twice as L1/L2 and S1/S2, respectively. Typical nematode tRNAs with a D-arm but no T-arm are indicated by (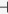), if they retained their T-arm and lack the D-arm are indicated by (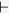). Structures lacking both the T-arm and the D-arm are denoted by (|). The intact clover-leaf structures are shown as (+). nf: not found.

**Table 5:** Non-coding regions longer than 30 bp found in the Longidoridae mitochondrial genomes.

*X. americanum*^a^			*X. rivesi*			*X. pachtaicum*			*L. vineacola*			*P. litoralis*		
Position	size	Genes/tRNA	Position	size	Genes/tRNA	Position	size	Genes/tRNA	Position	size	Genes/tRNA	position	size	Genes/tRNA
3943–4038*	96	*nad4L-nad3* (+)	8396–8433	37	Pro-Gln (−)	114–148	34	*Val-atp6* (+)	2839–3204	365	Ser2-Asn (+)	1035–1077	42	Gln-Pro (+)
12437–12472	36	Pro-Gln (−)	12529–12624*	96	*nad4L-nad3* (−)	821–858	37	Gly-Tyr (+/−)	3261–3391	130	Asn-*atp6* (+)	3252–3354*	103	*nad4L-nad3* (−)
12529–12575	47	Gln-Phe (−)				2887–2928	41	Gln-Pro (+)	4140–4263	123	Phe-Ala (+/−)	7040–7136	96	*nad4-Ser*1 (−)
						3571–3606	35	Asp-nad1 (−/+)	8314–8354	40	Pro-Gln (−)			
						7254–7393*	140	*nad4L-nad3* (−)	9824–9860	36	*nad5-nad6* (−)			
						9235–9274	39	*nad5-nad6*	11455–11511	56	Lys-*cob* (−/+)			
						9807–9848	41	Ile-*cox*II (+)	12929–13021*	93	*nad4L-nad3* (−)			

^a^Annotation position starts from *atp6*. In brackets is shown the sense in which the fragment is inserted: (+) sense strand; (−) antisense strand; (+/−) starts in sense and finished in antisense; (−/+) starts with antisense and finished with sense strand.

^*^CR: Replication Control Region.

**Table 6:** Longidoridae nematodes studied for their mitochondrial genome.

Species^a^	Sample code	Locality	Geographical Coordinates	Host plant	Primers Long-PCR	GenBank accession numbers
						Mitochondrial genome
***Xiphinema pachtaicum***	IAS	Córdoba, Córdoba province, Spain	37°51′37.28″ N, −4°47″3.27″ W	cultivated olive	5′-GGAACAGCAATAATTATAGTGGCAG-3 5′-GAGGATTAACTGGAATTGTTTTAGC-3′	KU746821
***Xiphinema rivesi***	Isla	Castillo de Locubín, Jaén province, Spain	37°32′26.49″ N, −3°57″22.58″ W	cherry tree	5′-GTTTACCGCTAGAAATCATAACAGC-3′ 5′-TTAGCTTCTTTTAGAGGGAGAAAGG-3′	KU746820
***Longidorus vineacola***	AR31	Tarifa, Cádiz province, Spain	36°03′49.5″ N, −5°40″18.2″ W	wild olive	5′-GCATGTCTTACTAGACCAAATCCTG-3′ 5′-TTCCAACAGGGATTAAAGTGTTTAG-3′	KU746818
***Paralongidorus litoralis***	Zahara	Zahara de los Atunes, Cádiz province, Spain	36°06′27.79″ N, −5°49″32.94″ W	*Pistacia lentiscus* L.	5′-TTTTAAGCCTATACAGCTTTGG-3′ 5′-AATGGCCTACTTTTTCCCCTACTAG-3′	KU746819

Species identifications were based on morphology and barcoding using D2-D3 expansion segments of 28S rDNA^a^.

^a^For species identification see^37^,^39^,^44^,^45^.
