# Supplementary material for: Mitochondrial genome diversity in dagger and needle nematodes (Nematoda: Longidoridae)
Source: Sci Rep. 2017 Feb 2;7:41813. doi: 10.1038/srep41813 (PMC5288807; doi:10.1038/srep41813)
Supplement: Supplementary Information [file srep41813-s1.pdf]

Supporting information

Mitochondrial genome diversity in dagger and needle nematodes (Nematoda: Longidoridae)

Palomares-Rius, J.E.[1], Cantalapiedra-Navarrete, C.[1], Archidona-Yuste, A.[1], Blok, V.C.[2], and Castillo, P.[1]

[1]Instituto de Agricultura Sostenible (IAS), Agencia Estatal Consejo Superior de Investigaciones Científicas (CSIC), Avda. Menéndez Pidal s/n, 14004 Córdoba, Spain.

[2]Cell and Molecular Sciences, The James Hutton Institute, Invergowrie, Dundee, DD2 5DA, United Kingdom. Correspondence and requests for materials should be addressed to J.E.P.R. (email: palomaresje@ias.csic.es)

Table S1. Models selected by Protest 3.2 and jModelTest 2.1.7 for protein coding genes (PCG) after GBLOCKS analysis. Selected models from the available programs in MrBayes 3.1.2 and RAxML 8.2.2 which best fit our dataset from the ranked models in Protest 3.2 and jModelTest 2.1.7.

Gene	Best model Protest 3.2	MrBayes 3.1.2	RAxML 8.2.2	Best model jModelTest 2.1.7	MrBayes 3.1.2	RAxML 8.2.2
atp6	MtArt	MtREV+F	MtArt	GTR+I+G	GTR+I+G	GTR+I+G
atp8	MtREV	MtREV	MtREV	HKY+I+G	HKY+I+G	GTR+I+G
cox1	MtREV+F	MtREV +F	MtREV+F	GTR+I+G	GTR+I+G	GTR+I+G
cox2	MtREV+F	MtREV+F	MtREV+F	GTR+I+G	GTR+I+G	GTR+I+G
cox3	MtREV+F	MtREV +F	MtREV+F	GTR+I+G	GTR+I+G	GTR+I+G
cytb	MtArt	MtREV+F	MtArt	GTR+I+G	GTR+I+G	GTR+I+G
nd1	MtArt	MtREV+F	MtArt	GTR+I+G	GTR+I+G	GTR+I+G
nd2	MtArt	MtREV+F	MtArt	GTR+I+G	GTR+I+G	GTR+I+G
nd3	MtREV+F	MtREV+F	MtREV+F	TVM+I+G	TVM+I+G	GTR+I+G
nd4	MtArt+F	WAG+F	MtArt+F	GTR+I+G	GTR+I+G	GTR+I+G
nd4l	MtArt	MtREV+F	MtArt	TVM+I+G	TVM+I+G	GTR+I+G
nd5	WAG+F	WAG+F	WAG+F	GTR+I+G	GTR+I+G	GTR+I+G
nd6	MtREV+F	MtREV+F	MtREV+F	TVM+I+G	TVM+I+G	GTR+I+G

Supporting Information Figures (Figs S1-S9)

Figure S1. Secondary structure models for tRNA genes (ALA, ARG, HIST, MET, PHE, CLY, LYS). Some genes were not found in some species. Xa: *Xiphinema americanum*, Xr: *X. rivesi*, Xp: *X. pachtaicum*, Pl: *Paralongidorus litoralis* and Lv: *Longidorus vineacola*.

Figure S2. Secondary structure models for tRNA genes (GLN, GLU, PRO, TRP, VAL, THR, TYR). Some genes were not found in some species. Xa: *Xiphinema americanum*, Xr: *X. rivesi*, Xp: *X. pachtaicum*, Pl: *Paralongidorus litoralis* and Lv: *Longidorus vineacola*.

Figure S3. Secondary structure models for tRNA genes (ASP, LEU1, LEU2, CYS, SER1, SER2, ASN). Some genes were not found in some species. Xa: *Xiphinema americanum*, Xr: *X. rivesi*, Xp: *X. pachtaicum*, Pl: *Paralongidorus litoralis* and Lv: *Longidorus vineacola*.

Figure S4. Stem–loop structures predicted for the noncoding region between the *nad4L* and *nad3* genes (replication control region, CR) for *Xiphinema americanum*, *Xiphinema rivesi* and *Xiphinema pachtaicum*.

Figure S5. Stem–loop structure predicted for the noncoding regions in *Xiphinema rivesi* and *X. pachtaicum*.

Figure S6. Stem–loop structure predicted for the noncoding regions in *Longidorus vineacola* and *Paralongidorus litoralis*.

Figure S7. Inferred phylogenetic tree resulting from Bayesian analysis of nucleotide sequences for 13 protein-coding genes from Enoplea and two Arthropoda outgroups. Bayesian probability values (BPP) shown above the node (≥70).

Figure S8. Single maximum likelihood tree with values from the separate bootstrap analysis and two Arthropoda outgroups. Analysis of nucleotide sequences for 13 protein-coding genes inferred using RAxML (see methods for analysis details). Bootstrap values shown above the node (≥70).

Figure S9. Partial 18S from Enoplea. Phylogenetic relationships between Enoplea species. Bayesian 50% majority rule consensus tree as inferred from the analysis of the partial 18S dataset under the GTR + I + G model. Posterior probabilities more than 70% are given for appropriate clades.

FIG. S1

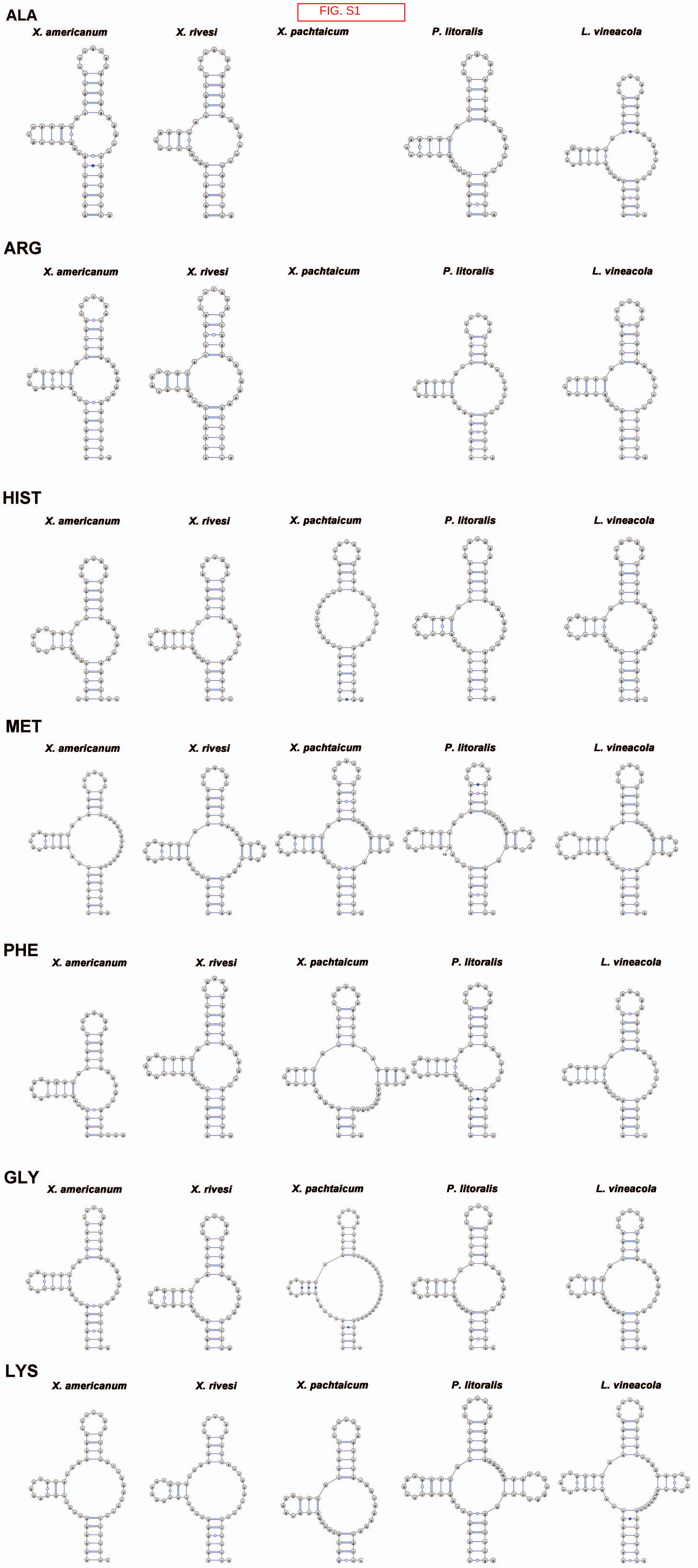
ALA
X. americanum
X. rivesi
X. pachtaicum
P. litoralis
L. vineacola
ARG
X. americanum
X. rivesi
X. pachtaicum
P. litoralis
L. vineacola
HIST
X. americanum
X. rivesi
X. pachtaicum
P. litoralis
L. vineacola
MET
X. americanum
X. rivesi
X. pachtaicum
P. litoralis
L. vineacola
PHE
X. americanum
X. rivesi
X. pachtaicum
P. litoralis
L. vineacola
GLY
X. americanum
X. rivesi
X. pachtaicum
P. litoralis
L. vineacola
LYS
X. americanum
X. rivesi
X. pachtaicum
P. litoralis
L. vineacola

FIG. S2

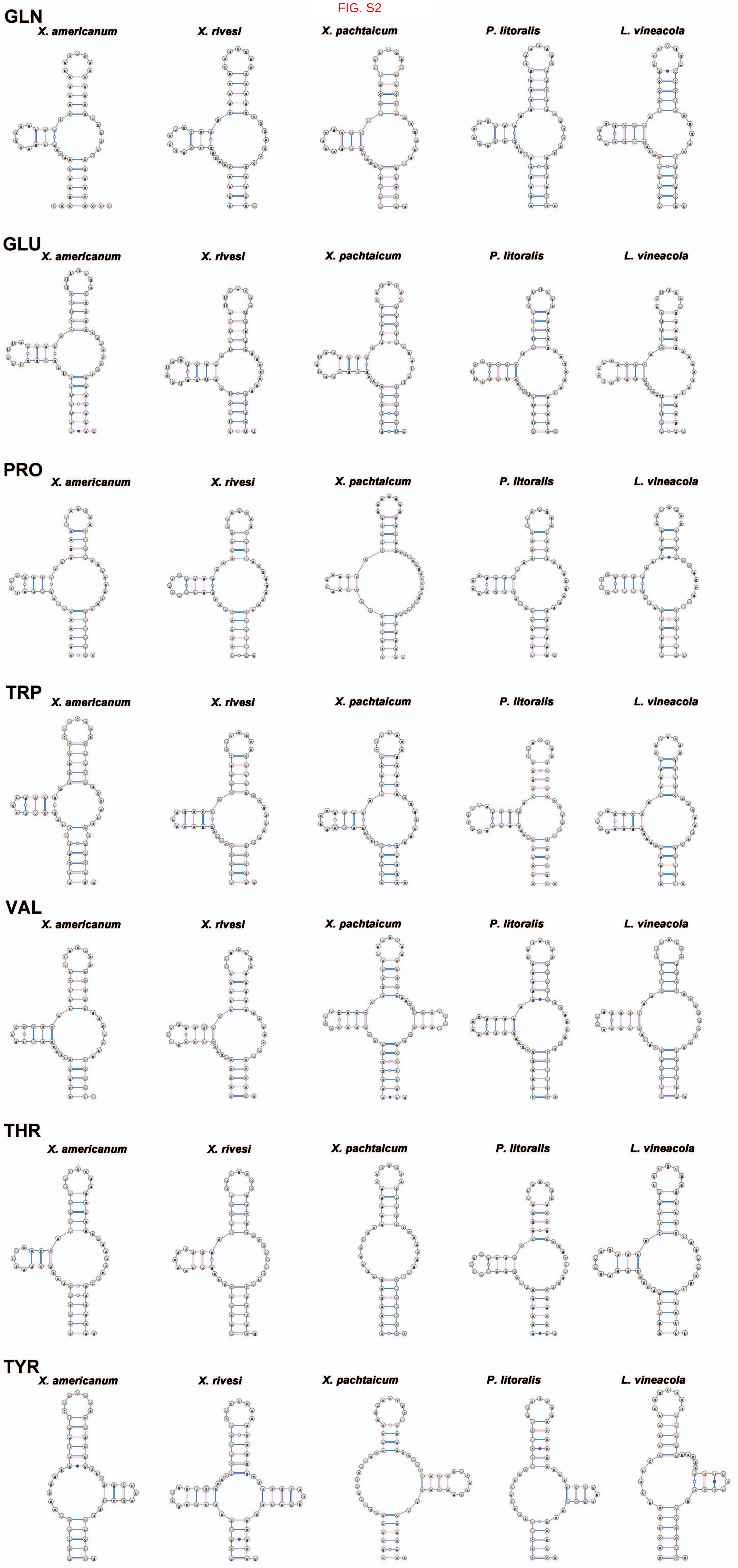
GLN
X. americanum
X. rivesi
X. pachtaicum
P. litoralis
L. vineacola
GLU
X. americanum
X. rivesi
X. pachtaicum
P. litoralis
L. vineacola
PRO
X. americanum
X. rivesi
X. pachtaicum
P. litoralis
L. vineacola
TRP
X. americanum
X. rivesi
X. pachtaicum
P. litoralis
L. vineacola
VAL
X. americanum
X. rivesi
X. pachtaicum
P. litoralis
L. vineacola
THR
X. americanum
X. rivesi
X. pachtaicum
P. litoralis
L. vineacola
TYR
X. americanum
X. rivesi
X. pachtaicum
P. litoralis
L. vineacola

FIG. S3

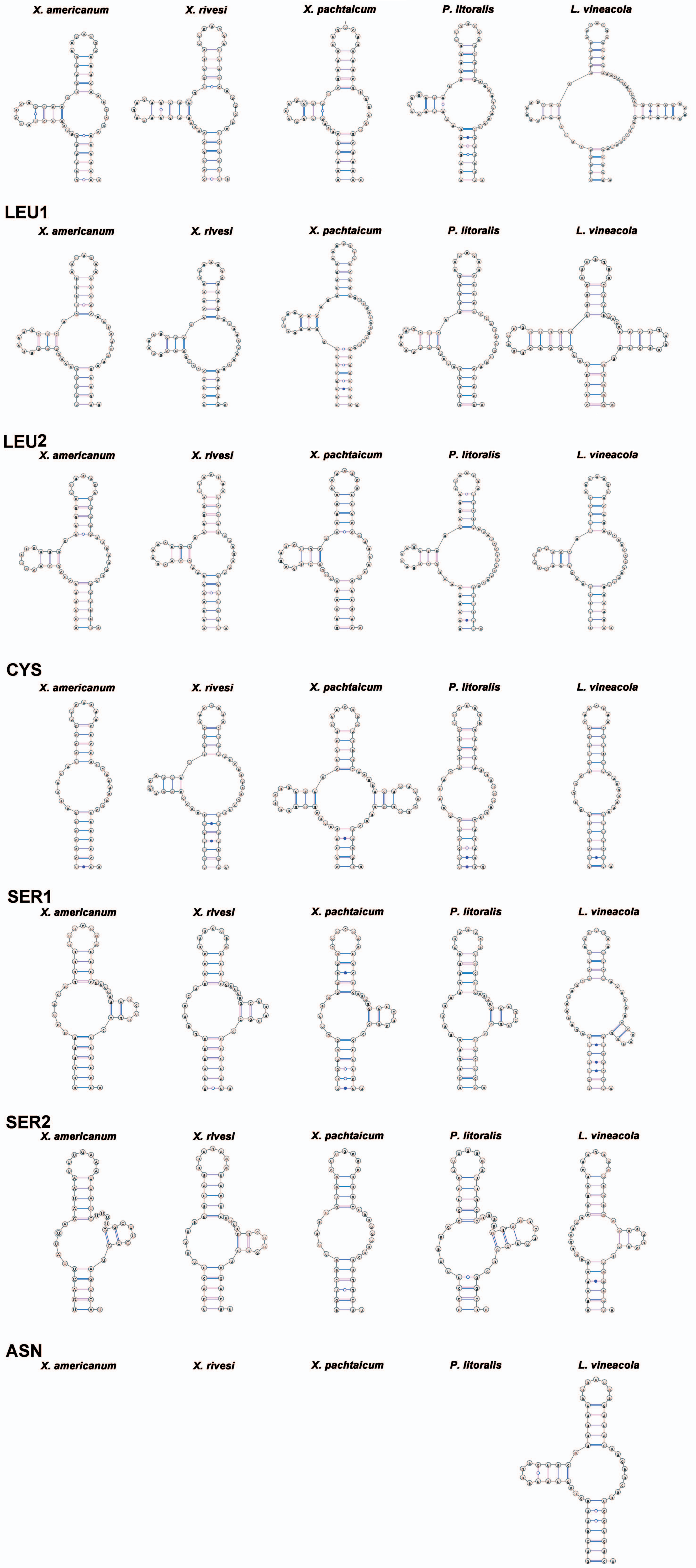
ASP
X. americanum
X. rivesi
X. pachtaicum
P. litoralis
L. vineacola
LEU1
X. americanum
X. rivesi
X. pachtaicum
P. litoralis
L. vineacola
LEU2
X. americanum
X. rivesi
X. pachtaicum
P. litoralis
L. vineacola
CYS
X. americanum
X. rivesi
X. pachtaicum
P. litoralis
L. vineacola
SER1
X. americanum
X. rivesi
X. pachtaicum
P. litoralis
L. vineacola
SER2
X. americanum
X. rivesi
X. pachtaicum
P. litoralis
L. vineacola
ASN
X. americanum
X. rivesi
X. pachtaicum
P. litoralis
L. vineacola

FIG. S4

CR *X. americanum*

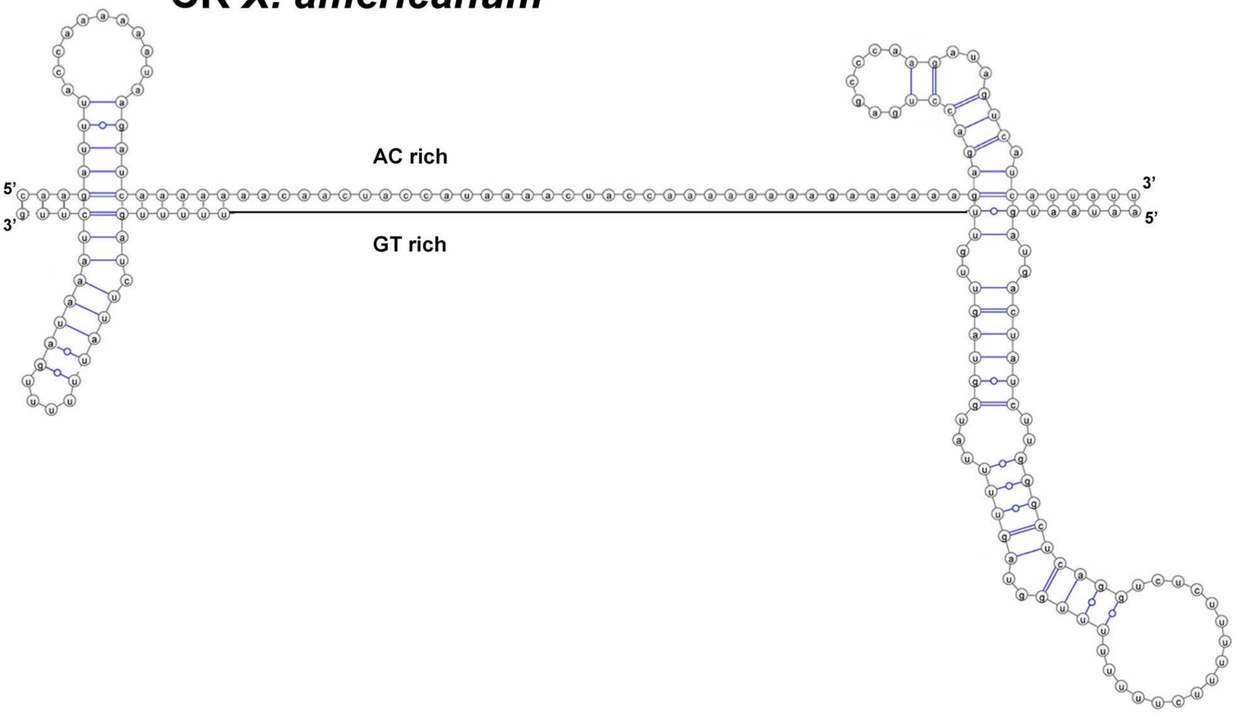

CR *X. rivesi*

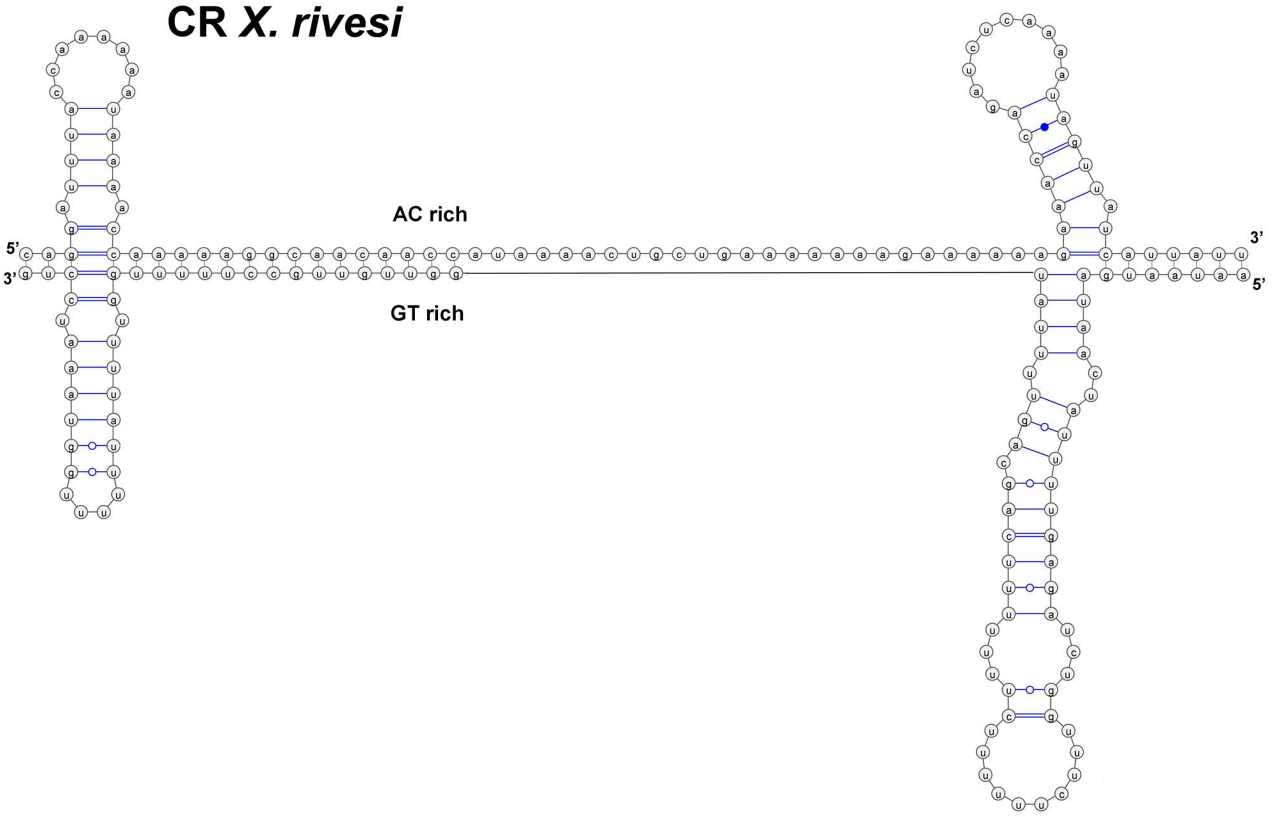

CR *X. pachtaicum*

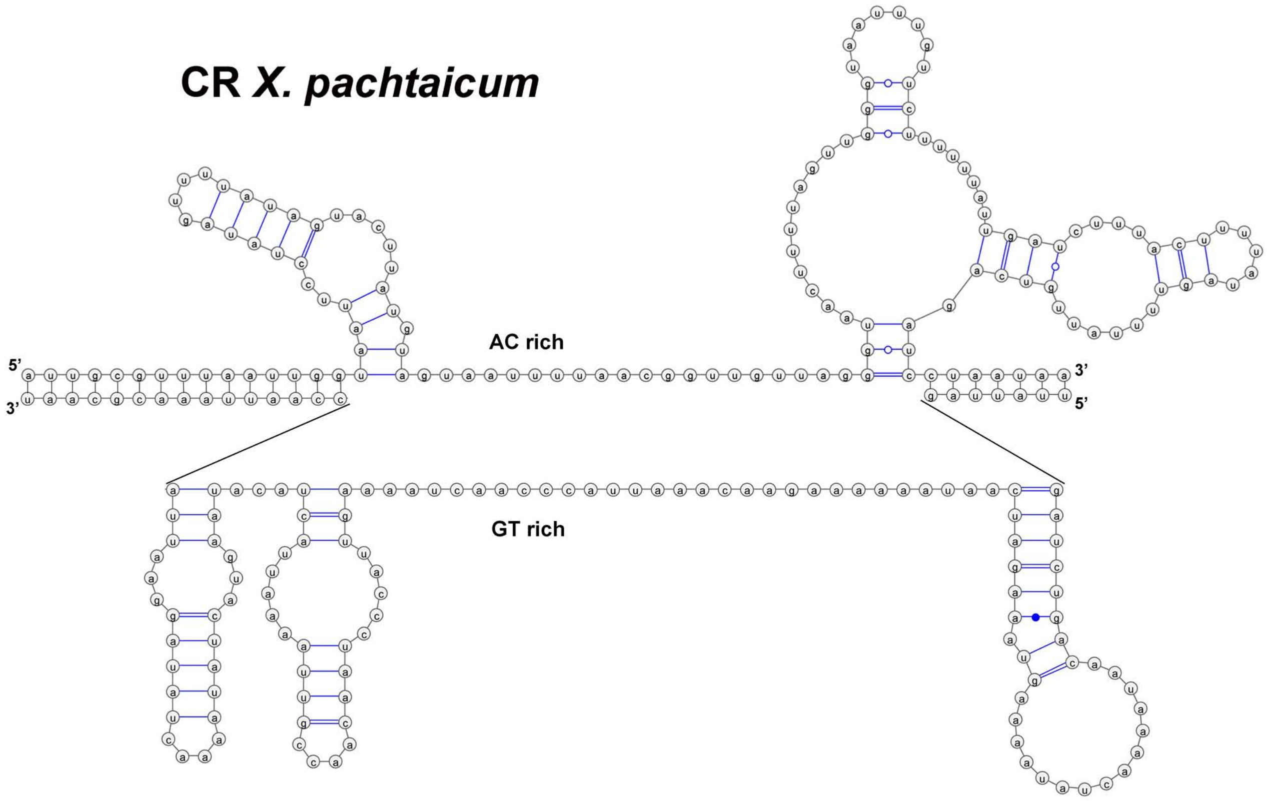

FIG. S5

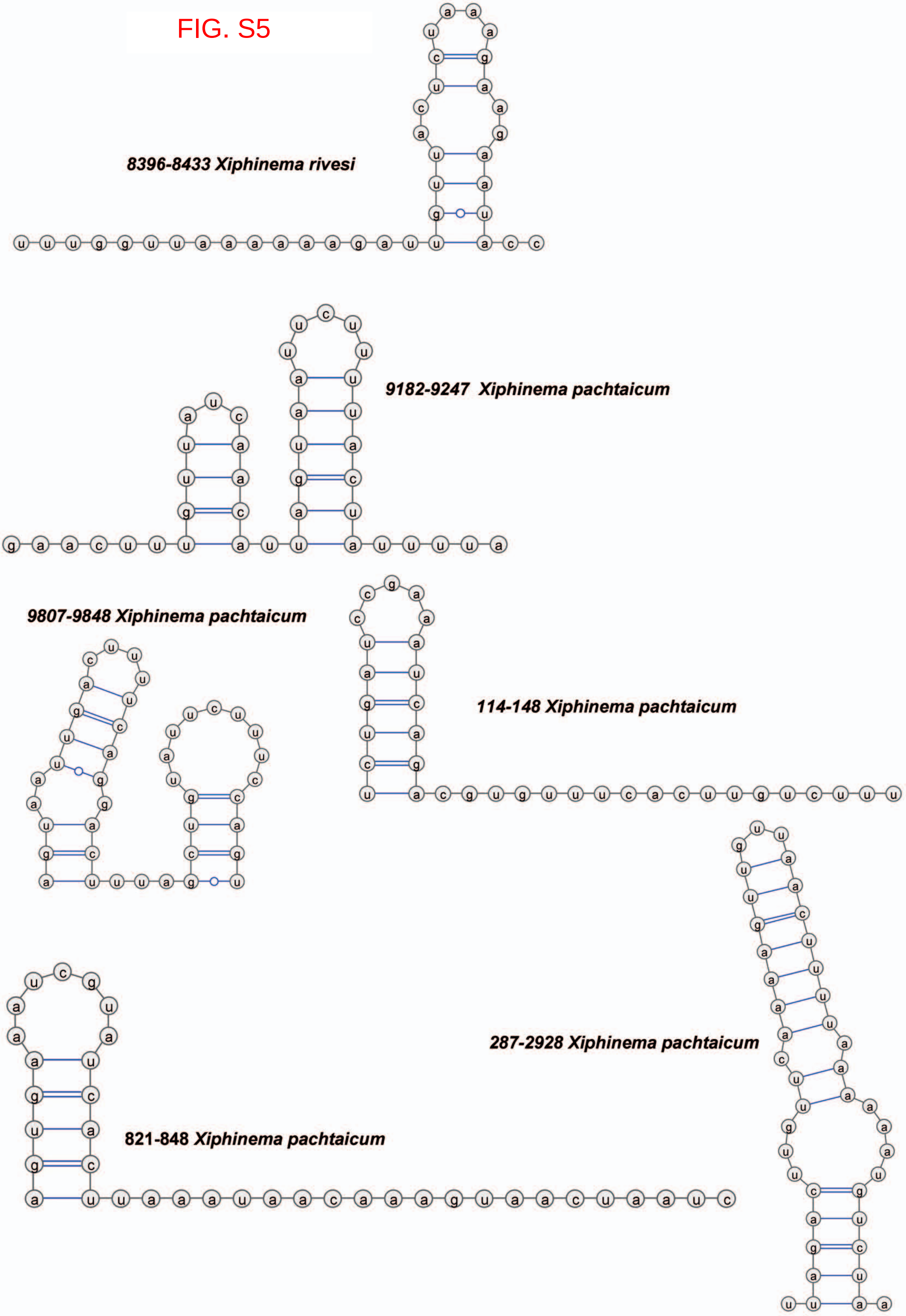
8396-8433 Xiphinema rivesi
9182-9247 Xiphinema pachtaicum
9807-9848 Xiphinema pachtaicum
114-148 Xiphinema pachtaicum
287-2928 Xiphinema pachtaicum
821-848 Xiphinema pachtaicum

FIG. S6

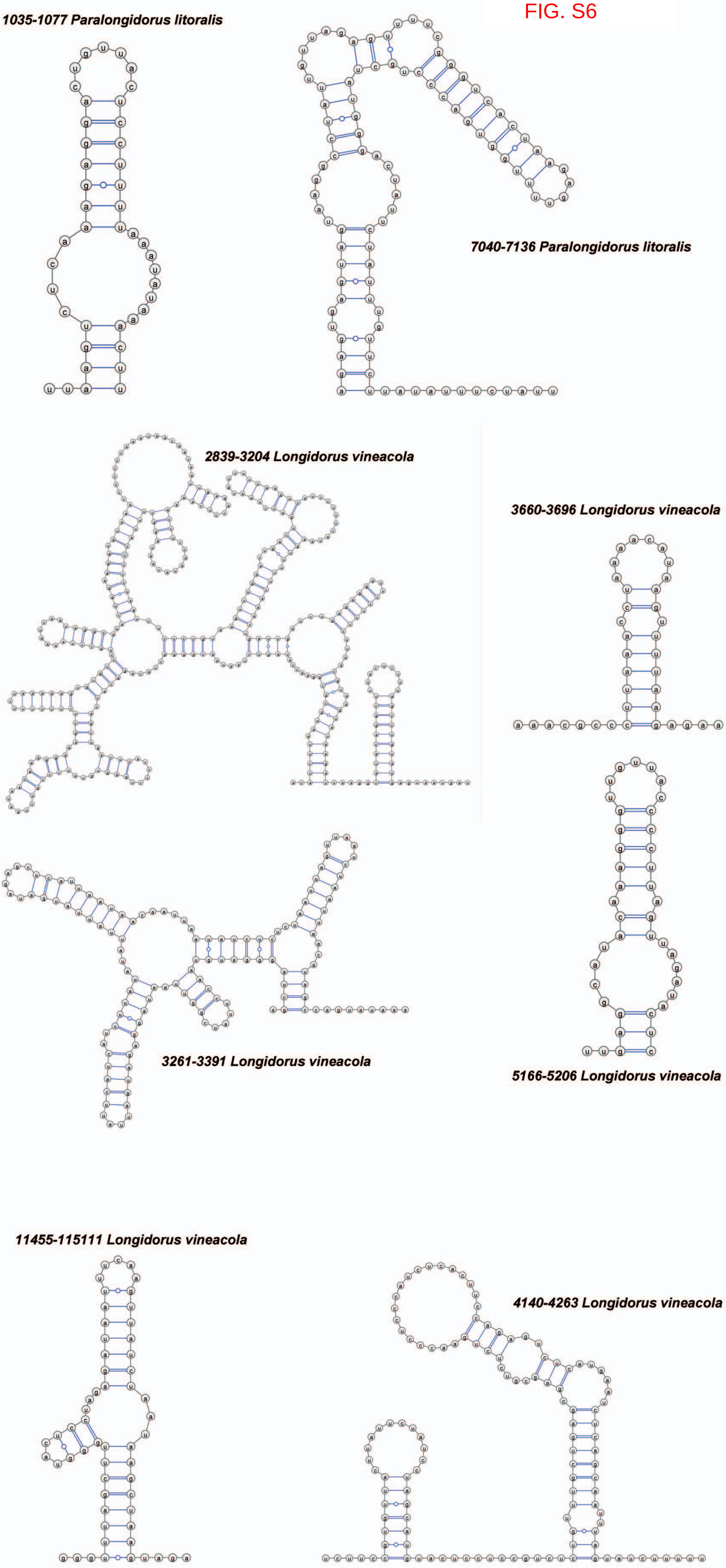

1035-1077 Paralongidorus litoralis
7040-7136 Paralongidorus litoralis
2839-3204 Longidorus vineacola
3660-3696 Longidorus vineacola
3261-3391 Longidorus vineacola
5166-5206 Longidorus vineacola
11455-115111 Longidorus vineacola
4140-4263 Longidorus vineacola

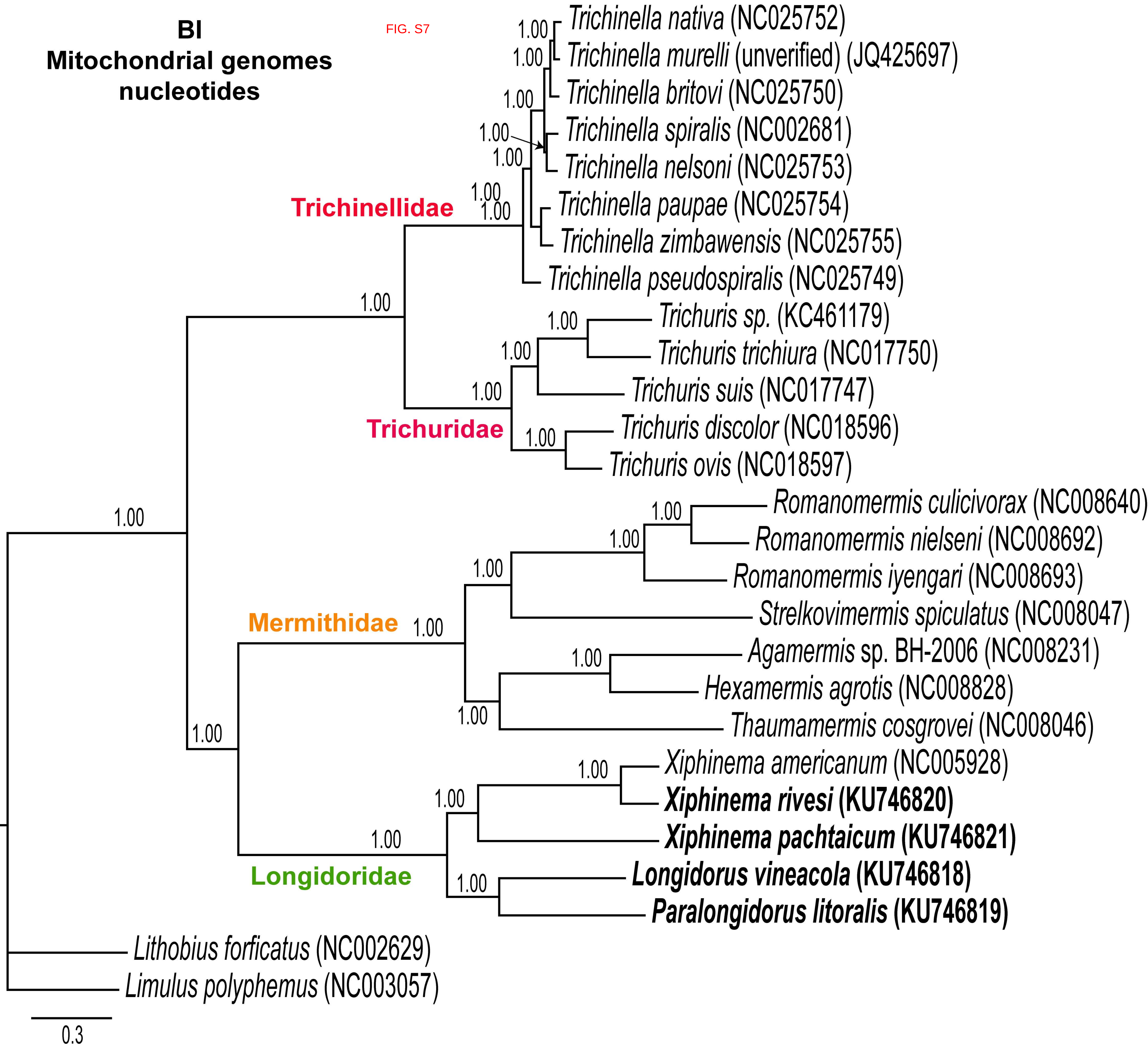
BI
Mitochondrial genomes
nucleotides
FIG. S7
Trichinellidae
Trichuridae
Mermithidae
Longidoridae
Trichinella nativa (NC025752)
Trichinella murelli (unverified) (JQ425697)
Trichinella britovi (NC025750)
Trichinella spiralis (NC002681)
Trichinella nelsoni (NC025753)
Trichinella paupae (NC025754)
Trichinella zimbawensis (NC025755)
Trichinella pseudospiralis (NC025749)
Trichuris sp. (KC461179)
Trichuris trichiura (NC017750)
Trichuris suis (NC017747)
Trichuris discolor (NC018596)
Trichuris ovis (NC018597)
Romanomermis culicivorax (NC008640)
Romanomermis nielseni (NC008692)
Romanomermis iyengari (NC008693)
Strelkovimermis spiculatus (NC008047)
Agamermis sp. BH-2006 (NC008231)
Hexamermis agrotis (NC008828)
Thaumamermis cosgrovei (NC008046)
Xiphinema americanum (NC005928)
Xiphinema rivesi (KU746820)
Xiphinema pachtaicum (KU746821)
Longidorus vineacola (KU746818)
Paralongidorus litoralis (KU746819)
Lithobius forficatus (NC002629)
Limulus polyphemus (NC003057)
1.00
0.3

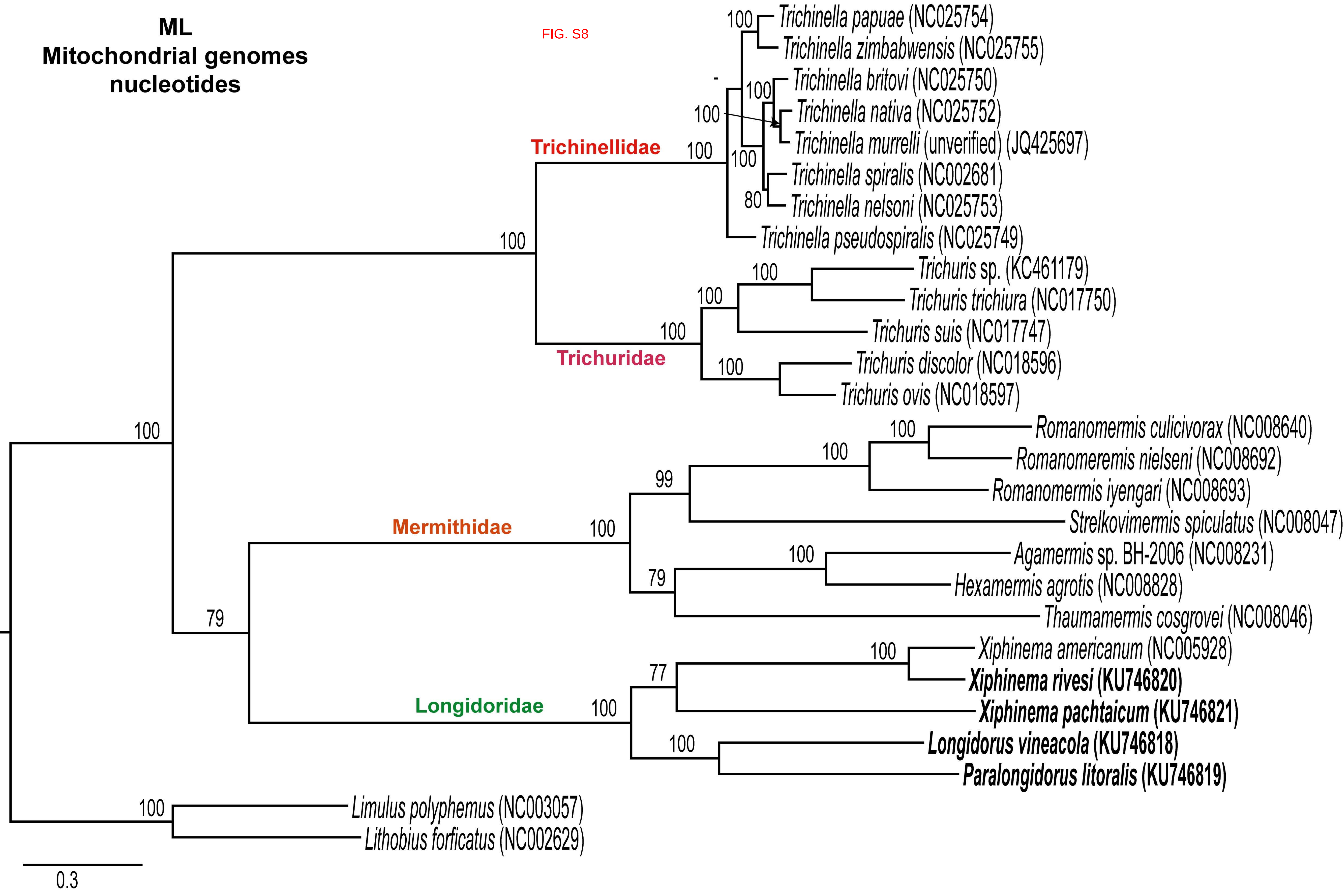
ML
Mitochondrial genomes
nucleotides
FIG. S8
Trichinellidae
Trichuridae
Mermithidae
Longidoridae
Trichinella papuae (NC025754)
Trichinella zimbabwensis (NC025755)
Trichinella britovi (NC025750)
Trichinella nativa (NC025752)
Trichinella murrelli (unverified) (JQ425697)
Trichinella spiralis (NC002681)
Trichinella nelsoni (NC025753)
Trichinella pseudospiralis (NC025749)
Trichuris sp. (KC461179)
Trichuris trichiura (NC017750)
Trichuris suis (NC017747)
Trichuris discolor (NC018596)
Trichuris ovis (NC018597)
Romanomermis culicivorax (NC008640)
Romanomeremis nielseni (NC008692)
Romanomermis iyengari (NC008693)
Strelkovimermis spiculatus (NC008047)
Agamermis sp. BH-2006 (NC008231)
Hexamermis agrotis (NC008828)
Thaumamermis cosgrovei (NC008046)
Xiphinema americanum (NC005928)
Xiphinema rivesi (KU746820)
Xiphinema pachtaicum (KU746821)
Longidorus vineacola (KU746818)
Paralongidorus litoralis (KU746819)
Limulus polyphemus (NC003057)
Lithobius forficatus (NC002629)
0.3

FIG. S9

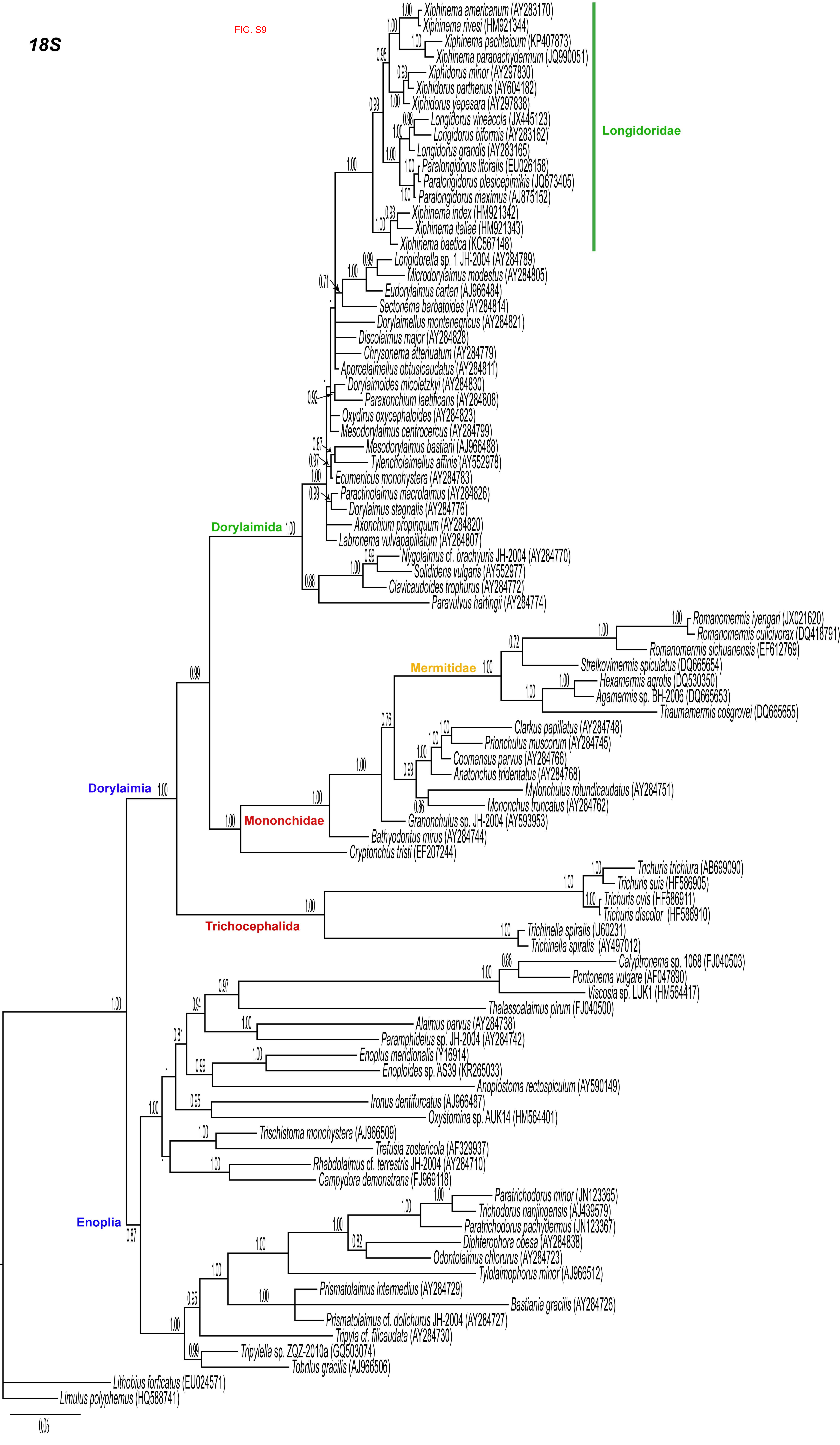
18S
Longidoridae
Xiphinema americanum (AY283170)
Xiphinema rivesi (HM921344)
Xiphinema pachtaicum (KP407873)
Xiphinema parapachydermum (JQ990051)
Xiphidorus minor (AY297830)
Xiphidorus parthenus (AY604182)
Xiphidorus yepesara (AY297838)
Longidorus vineacola (JX445123)
Longidorus biformis (AY283162)
Longidorus grandis (AY283165)
Paralongidorus litoralis (EU026158)
Paralongidorus plesioepimikis (JQ673405)
Paralongidorus maximus (AJ875152)
Xiphinema index (HM921342)
Xiphinema italiae (HM921343)
Xiphinema baetica (KC567148)
Longidorella sp. 1 JH-2004 (AY284789)
Microdorylaimus modestus (AY284805)
Eudorylaimus carteri (AJ966484)
Sectonema barbatoides (AY284814)
Dorylaimellus montenegricus (AY284821)
Discolaimus major (AY284828)
Chrysonema attenuatum (AY284779)
Aporcelaimellus obtusicaudatus (AY284811)
Dorylaimoides micoletzkyi (AY284830)
Paraxonchium laetificans (AY284808)
Oxydirus oxycephaloides (AY284823)
Mesodorylaimus centrocercus (AY284799)
Mesodorylaimus bastiani (AJ966488)
Tylencholaimellus affinis (AY552978)
Ecumenicus monohystera (AY284783)
Paractinolaimus macrolaimus (AY284826)
Dorylaimus stagnalis (AY284776)
Axonchium propinquum (AY284820)
Labronema vulvapapillatum (AY284807)
Nygolaimus cf. brachyuris JH-2004 (AY284770)
Solididens vulgaris (AY552977)
Clavicaudoides trophurus (AY284772)
Paravulvus hartingii (AY284774)
Dorylaimida
Mermitidae
Romanomermis iyengari (JX021620)
Romanomermis culicivorax (DQ418791)
Romanomermis sichuanensis (EF612769)
Strelkovimermis spiculatus (DQ665654)
Hexamermis agrotis (DQ530350)
Agamermis sp. BH-2006 (DQ665653)
Thaumamermis cosgrovei (DQ665655)
Clarkus papillatus (AY284748)
Prionchulus muscorum (AY284745)
Coomansus parvus (AY284766)
Anatonchus tridentatus (AY284768)
Mylonchulus rotundicaudatus (AY284751)
Mononchus truncatus (AY284762)
Granonchulus sp. JH-2004 (AY593953)
Bathyodontus mirus (AY284744)
Cryptonchus tristi (EF207244)
Mononchidae
Dorylaimia
Trichuris trichiura (AB699090)
Trichuris suis (HF586905)
Trichuris ovis (HF586911)
Trichuris discolor (HF586910)
Trichinella spiralis (U60231)
Trichinella spiralis (AY497012)
Trichocephalida
Calyptronema sp. 1068 (FJ040503)
Pontonema vulgare (AF047890)
Viscosia sp. LUK1 (HM564417)
Thalassoalaimus pirum (FJ040500)
Alaimus parvus (AY284738)
Paramphidelus sp. JH-2004 (AY284742)
Enoplus meridionalis (Y16914)
Enoploides sp. AS39 (KR265033)
Anoplostoma rectospiculum (AY590149)
Ironus dentifurcatus (AJ966487)
Oxystomina sp. AUK14 (HM564401)
Trischistoma monohystera (AJ966509)
Trefusia zostericola (AF329937)
Rhabdolaimus cf. terrestris JH-2004 (AY284710)
Campydora demonstrans (FJ969118)
Paratrichodorus minor (JN123365)
Trichodorus nanjingensis (AJ439579)
Paratrichodorus pachydermus (JN123367)
Diphterophora obesa (AY284838)
Odontolaimus chlorurus (AY284723)
Tylolaimophorus minor (AJ966512)
Prismatolaimus intermedius (AY284729)
Bastiania gracilis (AY284726)
Prismatolaimus cf. dolichurus JH-2004 (AY284727)
Tripyla cf. filicaudata (AY284730)
Tripylella sp. ZQZ-2010a (GQ503074)
Tobrilus gracilis (AJ966506)
Enoplia
Lithobius forficatus (EU024571)
Limulus polyphemus (HQ588741)
0.06
